# Punicic Acid in Ovarian Cancer: Anticancer Activity and Mechanistic Insights

**DOI:** 10.3390/cells15090792

**Published:** 2026-04-27

**Authors:** Jingjia Mo, Isabella Mendieta, Alexander J. Adams, Katherine Wiest, Hannah Lee, Victoria Gorman, Rachel Koo, Santiago Garcia, Ethan Nguyen, Aaron Lee, Jihua Feng, Zhiqing Huang

**Affiliations:** 1 Division of Reproductive Sciences, Department of Obstetrics and Gynecology, Duke University School of Medicine, Durham, NC 27701, USA; mojingjia@sr.gxmu.edu.cn (J.M.); bella.mendieta@duke.edu (I.M.); aj.adams@duke.edu (A.J.A.); katherine.wiest@duke.edu (K.W.); hannah.lee@duke.edu (H.L.); vicky.gorman@duke.edu (V.G.); rachel.koo@duke.edu (R.K.); santiago.garcia@duke.edu (S.G.); ethan.nguyen@duke.edu (E.N.); alee98@bu.edu (A.L.); fengjihua@gxmu.edu.cn (J.F.); 2 Department of General Practice, The Second Affiliated Hospital of Guangxi Medical University, Nanning 530007, China; 3 Department of Emergency, The Second Affiliated Hospital of Guangxi Medical University, Nanning 530007, China

**Keywords:** ovarian cancer, punicic acid, ferroptosis, mitochondrial dysfunction, free fatty acids, tumor microenvironment

## Abstract

**Highlights:**

**What are the main findings?**

- Punicic acid shows greater cytotoxicity in ovarian cancer cells than in normal cells and may enhance the effects of cisplatin.
- Punicic Acid induces ferroptosis, alters lipid metabolism, modulates mitochondrial function, and reprograms redox and ferroptosis-associated pathways in ovarian cancer cells.

**What are the implications of the main findings?**

- This study suggests that combining punicic acid with cisplatin may allow lower chemotherapy doses while maintaining or enhancing antitumor efficacy, potentially improving clinical tolerability.
- These findings propose mechanisms that may underly punicic acid’s preferential toxicity, providing insight into ovarian cancer cell vulnerabilities.

**Abstract:**

Ovarian cancer (OC) remains the deadliest gynecological malignancy, with aged tumor microenvironments linked to poorer outcomes. Our prior work identified reduced levels of free fatty acids (FFAs) within tumor-surrounding adipose tissue of aged OC xenograft rats compared to younger counterparts. In this study, we investigated the therapeutic potential of one such FFA, punicic acid (PunA). We evaluated PunA’s effects on OC and normal cell viability and compared its activity with that of its structural isomer, α-eleostearic acid (α-ESA). Both compounds decreased OC cell viability; however, α-ESA was cytotoxic to normal cells, whereas PunA selectively impaired OC cell viability while sparing normal cells. Additionally, PunA enhanced cisplatin efficacy, demonstrating its potential for use in combination therapy to reduce cisplatin dosage and toxicity without compromising antitumor activity. Mechanistically, PunA induced ferroptosis in OC cells while sparing normal cells by differently modulating lipid peroxidation, fatty acid oxidation, and mitochondrial function. Transcriptomic profiling further revealed coordinated gene expression changes associated with oxidative stress and ferroptosis in PunA-treated OC and normal cells. In a preliminary C57BL/6J-ID8 OC mouse model, PunA suppressed tumor growth. Collectively, these findings identify PunA as a promising therapeutic candidate for OC, acting through ferroptosis and mitochondrial dysfunction, and enhancing cisplatin efficacy while sparing normal cells.

## 1. Introduction

Ovarian cancer (OC) is one of the leading causes of cancer-related deaths in women, with a lifetime risk factor of approximately 1 in 91 [1,2]. In its early stages, OC does not exhibit specific symptoms and is thus mostly diagnosed when it has already reached more advanced stages with aggressive metastasis in the peritoneal cavity and even distant organs [3]. Most OC cases occur in older women, with a median age at diagnosis of 63 years [2]. In addition, cancer recurrence and metastasis, which are among the most common complications for OC, often increase with age [4]. The combination of many factors, such as aging, lack of effective diagnostic strategies, late detection, development of resistance to therapy, and frequent recurrence, leads to a relatively high mortality rate in OC patients [3]. Continued research is urgently needed to identify therapeutic strategies that not only target the aggressive and resistant nature of OC but also address age-related changes in tumor biology to improve early detection and treatment outcomes.

Aging significantly impacts OC progression and metastasis, primarily through alterations in both the systemic macroenvironment and the tumor microenvironment (TME) [5,6]. The TME consists of both non-cellular elements, such as the extracellular matrix (ECM), and cellular components—including immune cells, fibroblasts, endothelial cells, and adipocytes—all of which create a more permissive environment for cancer cell survival, growth, and spread [6]. Adipose tissue (AT), also referred to as the adipose tumor microenvironment (AME), represents a major component of the TME in OC and plays a significant role in promoting both cancer initiation and disease progression [7,8]. OC begins its progression in the peritoneal cavity, subsequently spreading to the AT within the intraperitoneal cavity, specifically in the omentum [9]. The interaction between the OC cells and the AT in the omentum induces morphological changes in adipocytes, leading to the formation of cancer-associated adipocytes (CAAs) [10]. These adipocytes contribute to the progression of OC through multiple mechanisms, including tumor growth, chemoresistance, metastasis, and even recurrence [11]. The impact of adipocytes on OC is thus an important direction of research, especially because AT is one of the earliest organs to change in response to aging [12]. It was largely unknown, however, the extent to which these age-related changes impact OC cells, OC progression, and chemotherapies.

To address this gap, we investigated the roles of aging on the adipocyte-rich TME in an OC xenograft rat model through lipidomic and transcriptomic profiling of tumor-surrounding AT [13]. In this previous study, our findings demonstrated that aged RNU nude rats (12 months old) were more susceptible to and exhibited more aggressive tumor development, with 100% (13 out of 13 rats) of the aged rats forming tumors, compared to 68.75% (11 out of 16 rats) of the young rats (6 weeks old). Median tumor volumes of 17.6 cm^3^ and 1.1 cm^3^ were observed in the aged and younger cohorts, respectively. Using tumor-surrounding AT tissues from aged OC rats, RNA sequencing analysis revealed notable upregulation of genes associated with inflammation and lipid regulation, including *S100a8*, *S100a9*, *Lcn2*, *C3*, and *Pnpla3*, when compared to the AT from young OC rats. In addition, aged OC ATs exhibited significantly higher (*p* < 0.05) infiltration of neutrophils, dendritic cells, and CD^4+^ T-cells (non-regulatory) compared to their ATs before tumor formation, reflecting pro-inflammatory shifts that are conducive to cancer progression. These immune alterations were strongly correlated with changes in *S100a8*/*a9* expression and lipid levels.

Lipidomic profiling further revealed a significant downregulation (*p* < 0.05) of multiple free fatty acid (FFA) species in tumor-surrounding AT from aged OC rats compared to AT from their younger counterparts [13]. Among these, an FFA 18:3 (omega-5), chemically designated (9E,11Z,13E)-octadecatrienoic acid and more commonly known as punicic acid (PunA), showed the most pronounced decrease [13]. Interestingly, preliminary in vitro studies demonstrated that PunA diverged from other lipid alterations by exhibiting antitumor properties, as it significantly inhibited viability across three OC cell lines: A2780, HEYA8, and CAOV2 (*p* < 0.001) [13].

Building on this finding, the current study investigates the chemotherapeutic potential of FFAs in OC with a focus on PunA. We also examined another FFA 18:3 omega-5 member, alpha-eleostearic acid (α-ESA), because these two conjugated trienoic fatty acids share nearly identical molecular structures, differing only in the geometric arrangement of the double bond at the omega-5 position. PunA contains Δ9 cis, Δ11 trans, and Δ13 cis double bonds, while α-ESA possesses Δ9 cis, Δ11 trans, and Δ13 trans double bonds. As essential nutrients, neither compound can be synthesized endogenously and must be obtained through the diet [14]. PunA is derived primarily from pomegranate (*Punica granatum*) seed oil, while α-ESA is found predominantly in tung (*Aleurites fordii*) and bitter ground (*Momordica charantia*) seed oils [15]. Both compounds have garnered considerable attention due to their reported health benefits.

PunA, in particular, is known for its antidiabetic, anti-obesity, anti-inflammatory, and anticancer properties [14]. Nugteren et al. demonstrated that PunA inhibits cyclooxygenase activity, thereby reducing prostaglandin synthesis and contributing to its anti-inflammatory effects [16]. Using in vitro cancer models, PunA has been shown to reduce the viability of LNCaP (prostate cancer) cells at concentrations above 10 μM through pro-apoptotic and growth-inhibitory pathways [17]. In MDA-MB-231 and MDA-ERα7 (breast cancer) cell lines, a 40 μM dose of PunA reduced cell growth by over 90% and triggered apoptosis in more than 85% of the cells [18]. Notably, Quitmeyer et al. reported that MCF-10 (non-tumorigenic breast epithelial) cells were more sensitive to PunA-induced cytotoxicity than breast cancer MCF-7 cells at low concentrations [19].

The anticancer activity of α-ESA has also been demonstrated across a range of human cancer cell lines, where it exhibits dose-dependent cytotoxic effects in DLD-1 (colorectal cancer), HepG2 (liver cancer), and A549 (lung cancer) cells [20]. In breast cancer MCF-7 cells, Zhang et al. have demonstrated that α-ESA upregulates several pro-apoptotic markers, including PPAR-γ, p21, Bax, p53, and caspase-3 [21].

In the context of OC, natural compounds related to PunA and α-ESA have demonstrated anticancer effects. For example, pomegranate fruit juice and two of its principal constituents, ellagic acid and luteolin, suppress OC proliferation, migration, and progression by downregulating expression of MMP-2 and MMP-9 in A2780 cells (ovarian cancer) and nude mouse models [22]. Similarly, bitter melon and its bioactive compounds, such as kuguacin J, inhibit OC by inducing apoptosis, suppressing proliferation and migration, altering cancer cell metabolism, and enhancing the efficacy of chemotherapy drugs, all with minimal toxicity in preclinical models [23,24]. Despite the promising findings for related compounds, no studies have evaluated the anticancer potential of PunA and α-ESA in OC, including their cytotoxic effects on tumor-surrounding normal cells or the underlying mechanisms involved. Evidence from their efficacy in other cancers underscores the need to address this gap to determine whether PunA and α-ESA share, or even surpass, the therapeutic properties of their structurally related counterparts.

We previously demonstrated that FFAs are downregulated in aged OC xenografts and that certain FFA compounds may possess anticancer activity [13]. In the present study, we investigated the therapeutic potential of two structurally related FFAs, PunA and α-ESA, in treating OC, a disease highly prevalent in aged women and recognized as an age-associated cancer. Given their close structural similarity and prior evidence of anticancer properties, we directly compared the effects of PunA and α-ESA. To assess cell-specificity and potential cytotoxicity, we tested normal ovarian epithelial cells, normal fallopian tube (NFT) cells, and adipocytes commonly found in the tumor-surrounding AT. Because chemoresistance is a major challenge in OC, we also studied the effects of PunA on chemo-resistant OC cells. In addition, we investigated how fats, as a major component of OC tissues, influence PunA’s chemotherapeutic effects. To better understand the differential functions of PunA in cancer and normal cells, we conducted an in-depth study of mechanisms involving peroxisome proliferator-activated receptor gamma (PPARγ), ferroptosis, lipid peroxidation, and mitochondrial function. We further explored gene expression profiling and molecular pathways through which PunA may exert its effects. A clearer understanding of PunA’s functions will clarify how, in the context of OC, it preferentially targets cancer cells while sparing normal cells. This knowledge is crucial for opening new avenues in the development of effective, low-toxicity chemotherapeutic treatments for OC patients.

## 2. Materials and Methods

### 2.1. Cell Lines and Reagents

The OC cell lines HEYA8 and CAOV2 were obtained from the Duke Gynecologic Oncology Cell Line Repository (Durham, NC, USA). The OC cell lines A2780, A780cisR, 41M, and 41McisR were obtained from Kyoto University (Kyoto, Japan). OC cells were maintained in RPMI 1640 medium (Cat # A4192301, Thermo Fisher, Norristown, PA, USA) supplemented with 10% fetal bovine serum (FBS; Cat # A5256801, Thermo Fisher, Norristown, PA, USA) and 1% penicillin-streptomycin (P/S; Cat #P4333, MilliporeSigma, Darmstadt, Germany). The mouse preadipocyte 3T3-L1 cell line (Cat # CL-173) was purchased from the American Type Culture Collection (ATCC; Manassas, VA, USA). 3T3-L1 cells were cultured in DMEM with high glucose (Cat # D5796, MilliporeSigma, Darmstadt, Germany), supplemented with 10% bovine calf serum (BCS; Cat # 12133C, MilliporeSigma, Darmstadt, Germany) and 1% P/S. Mouse ID8 ovarian carcinoma cells stably expressing firefly luciferase (ID8-Luc) were generously provided by Dr. Ashley Chi (Duke University, Durham, NC, USA). ID8-Luc cells were maintained in DMEM with high glucose supplemented with 10% BCS and 1% P/S. Following the administration of luciferin substrate, luciferase expression enables bioluminescence imaging and allows tumor detection using the IVIS imaging system in vivo. Normal ovarian surface epithelial cells (HOSE-E7:hTERT; referred to as E7) were obtained from Kumamoto University (Kumamoto, Japan). The P201 and P211 NFT cell lines, immortalized with SV40 and hTERT, were obtained from Dr. Jazaeri (University of Virginia, Charlottesville, VA, USA) [25]. E7, P201, and P211 cells were cultured in RPMI 1640 supplemented with 10% FBS and 1% P/S. All cell lines were incubated at 37 °C in a humidified chamber with 5% CO_2_. Cell line authentication was performed by the Duke University DNA Analysis Facility to verify their identity, purity, and authenticity. All cell lines were tested and confirmed to be free of mycoplasma contamination by the Duke Cell Culture Facility.

Punicic acid (PunA; 9Z,11E,13Z-octadecatrienoic acid; Cat # 26057) and α-eleostearic acid (α-ESA; 9Z,11E,13E-octadecatrienoic acid; Cat # 10008349) were purchased from Cayman Chemical (Ann Arbor, MI, USA). Cisplatin (cis-diamminedichloroplatinum (II); Cat # HY-17394) and Ferrostatin-1 (Fer-1; Cat # HY-100579) were purchased from MedChemExpress (Monmouth Junction, NJ, USA).

### 2.2. Cell Treatments

#### 2.2.1. PunA, α-ESA, and Cisplatin Treatment of OC and Normal Cell Lines

The OC cell line HEYA8 (2000 cells/well) and normal cell lines NFT P201 (2000 cells/well), NFT P211 (2000 cells/well), ovarian epithelial E7 (4000 cells/well), and mouse preadipocyte 3T3-L1 (5000 cells/well) were seeded into 96-well plates after reaching ~70% confluency. Cells were cultured in the aforementioned medium. Cell viability and counts were determined prior to seeding using a hemocytometer and Trypan Blue staining (Cat # 15250061, Thermo Fisher, Norristown, PA, USA). After 24 h incubation at 37 °C with 5% CO_2_, cells were treated with vehicle control (methanol; Cat # BDH1135-4LP, VWR, Radnor, PA, USA), varying concentrations of PunA or α-ESA, or 20 μM cisplatin. PunA was initially dissolved in methanol to prepare a stock solution (10 mg/mL) and subsequently diluted in culture medium. The final methanol concentration ranged from approximately 0.04% to 0.44% (*v*/*v*), corresponding to PunA concentrations of 15–160 μM, with the highest concentration associated with the maximum PunA dose. Vehicle controls were prepared to match the highest methanol concentration corresponding to the maximum PunA dose in each experiment, providing a conservative control for potential solvent effects. Each treatment condition was performed in six technical replicates, and experiments were independently repeated at least three times.

#### 2.2.2. Combinational Chemo-Treatment

The OC cell line HEYA8 (2000 cells/well) was treated with PunA alone, cisplatin alone, or a combination of PunA + cisplatin. The seeding density and drug concentrations were determined based on preliminary optimization experiments. Treatment groups consisted of vehicle control (methanol), 15 μM PunA, 30 μM PunA, 30 μM cisplatin, and 30 μM PunA + 30 μM cisplatin. PunA was dissolved in methanol and further diluted in culture medium, and the vehicle control contained the same methanol concentration as the highest PunA concentration. The treatment was performed for 48 h, followed by an MTS assay to assess cell viability relative to control. Each treatment condition was tested in six technical replicates, and the experiment was performed in two biological replicates.

#### 2.2.3. PunA Treatment on Cisplatin-Resistant Cell Lines

The experiment was performed separately with two parental OC cell lines, 41M and A2780, and their cisplatin resistant pairs, 41M-cisR and A2780-cisR. Each cell line was cultured in RPMI medium supplemented with 10% FBS and 1% P/S until reaching approximately 80% confluency, then seeded into 96-well plates, at 8000 cells/well for M41 and 41M-cisR, and 3000 cells/well for A2780 and A2780-cisR. After 24 h of incubation, the cells were treated with PunA at concentrations ranging from 0 μM to 120 μM for 48 h at 37 °C with 5% CO_2_. PunA was dissolved in methanol and further diluted in culture medium, and the vehicle control contained the same methanol concentration as the highest PunA treatment condition. An MTS assay (as described below) was performed to determine cell viability and to compare the parental and cisR cell pairs using the method indicated in the statistical analysis. Each treatment condition was tested in six technical replicates per experiment, and the experiment was performed in three biological replicates.

#### 2.2.4. PunA Treatment on OC Cells in High Versus Low Fat Medium

The high-fat conditioned medium was generated from differentiated (DF) 3T3-L1 preadipocytes following an established protocol from Abcam (Cat # ab287843, Cambridge, UK). This protocol uses a “differentiation cocktail” containing insulin, dexamethasone, and 3-isobutyl-1-methylxanthine (IBMX), followed by a maintenance medium with a lower concentration of these components. The medium from the undifferentiated (UD) 3T3-L1 was used as low-fat condition control. HEYA8 cells were maintained in RPMI medium supplemented with 10% FBS and 1% penicillin-streptomycin (P/S) until reaching approximately 80% confluency, then seeded at 3000 cells per well into 96-well plates and incubated for 24 h at 37 °C with 5% CO_2_. HEYA8 cells were then treated with PunA at concentrations ranging from 0 to 100 μM in either DF or UD medium conditions and incubated for 48 h. PunA was dissolved in methanol and further diluted in the respective media, and vehicle controls contained the same methanol concentration as the highest PunA treatment condition. Cell viability was measured by MTS assay to evaluate resistance to PunA in high fat versus control media. Each treatment condition was tested in six technical replicates per experiment, and the experiment was performed in three biological replicates.

#### 2.2.5. Ferrostatin-1 Treatment

The OC cell lines HEYA8 and A2780 were cultured to approximately 70% confluency and seeded into 96-well plates at 2000 cells/well for HEYA8 and 3000 cells/well for A2780. After 24 h of incubation at 37 °C with 5% CO_2_, cells were treated with vehicle control (methanol), 2 µM of Fer-1, PunA, and Fer-1 + PunA. PunA was dissolved in methanol and further diluted in culture medium, and the vehicle control contained the same methanol concentration as the highest PunA condition. PunA was used at 25 µM for HEYA8 cells and 30 µM PunA for A2780 cells. Cells were treated for 48 h prior to assessment of viability using the MTS assay (as described below). Each treatment condition was tested in six technical replicates per experiment, and the experiment was performed in four biological replicates.

### 2.3. Cell Viability Assay (MTS Assay)

The CellTiter 96^®^ AQueous One Solution Cell Proliferation Assay was performed with 3-(4,5-dimethylthiazol-2-yl)-5-(3-carboxymethyl phenyl)-2-(4-sulfophenyl)-2H-tetrazolium (Cat # G3582, Promega, Durham, NC, USA), a colorimetric method (MTS Assay) for determining cell viability. After the specified incubation period for each test, 20 µL of the MTS solution was added to each well of the 96-well microplates, each containing 100 µL of a mixture of cells and treatment medium. Following a 1–4 h incubation at 37 °C, the absorbance for cell viability was measured at 490 nm using the FluoStar Omega Plate Reader (BMG Labtech, Ortenberg, Germany). 

### 2.4. Lipid Peroxidation Assay by Flow Cytometry

The OC cell line HEYA8 and the NFT epithelial cell line P201 were used for this test. HEYA8 and P201 cells at 3 × 10^5^ were seeded into each well of 6-well plates and incubated in RPMI 1640 medium with 10% FBS and 1% P/S at 37 °C and 5% CO_2_ for 24 h. Following incubation, the cells were divided into three treatment groups: untreated control, vehicle control (methanol at the same final concentration as in the PunA-treated group), and 10 µM PunA treatment. All groups were incubated for 48 h before BODIPY™ 581/591 C11 (Cat # D3861, Thermo Fisher, Norristown, PA, USA) assay. This treatment duration was specifically chosen to allow enough time for PunA to be incorporated into cellular membranes and have its hypothesized effects on lipid metabolism and oxidation. After 48 h of treatment, the HEYA8 and P201 cells were incubated in 10 µM concentration of BODIPY™ 581/591 C11 undecanoic acid (Lipid Peroxidation Sensor) within the medium for 30 min at 37 °C. This sensor probe undergoes a shift upon oxidation, which allows for the detection and quantification of lipid peroxidation in live cells. After incubation, the media was removed, and the cells were washed with Dulbecco’s Phosphate-Buffered Saline (PBS). The cells were collected using Trypsin in 15 mL tubes and washed with PBS three times, centrifuging for 5 min at 2000 RPM after each wash. After the third wash, 1 mL of PBS was added to resuspend the cells, and the cells were filtered through the mesh attached to the flow cytometry tubes to ensure single-cell suspension. The cells were imaged using the BD LSRFortessa X-20 machine (BD Biosciences, San Jose, CA, USA) within 2 h of staining to maintain signal integrity, and the fluorescence was read at two separate wavelengths: one at excitation/emission of 581/591 nm (Texas Red^®^ filter set) to detect the reduced dye, and the other at excitation/emission of 488/510 nm (traditional FITC filter set) to detect the oxidized dye. This test was repeated twice for both cell lines to quantitatively assess the shift from Texas Red to Green FITC which indicates lipid peroxidation in response to different treatment conditions. The two tests were analyzed using Student’s *t*-test to compare lipid peroxidation levels under PunA treatment versus vehicle control.

### 2.5. Fatty Acid Oxidation (FAO) Assay

Four cell lines, including two OC cell lines, A2780 and HEYA8, and two noncancerous cell lines, E7 and P201, were cultured in RPMI 1640 Medium with 10% FBS and 1% P/S at 37 °C and 5% CO_2_. Once the cells reached 80% confluence, they were split into 10 cm Petri dishes (P10) for treatment, with approximately 1 × 10^6^ cells per dish per cell line. The cells were then treated with PunA or vehicle control, with methanol present at the same final concentration as in the corresponding PunA-treated samples. For the HEYA8 cell line, a 10 µM treatment of PunA was used. For the A2780, E7, and P201 cell lines, 25 µM treatments of PunA were used. Treatment concentrations were chosen based on our studies on cell viability using varying PunA doses. The treatment was administered 6 h after cell seeding. 48 h post-treatment, the adherent cells were dissociated using trypsin and neutralized with RPMI + 10% FBS growth medium. Cells were collected and pelleted at 3000 rpm for 5 min in a 4 °C centrifuge. The following steps were then performed with the Fatty Acid Oxidation Kit (Cat # ab118183, Abcam, Cambridge, UK) according to the procedure from the manufacturer. Briefly, the cells were resuspended in PBS to a concentration of 2–5 × 10^6^ cells/mL. Fixation was performed using 4% paraformaldehyde at room temperature for 15 min. Fixed cells were then incubated in a 1× Permeabilization Buffer (0.1% Triton X-100 in PBS) for 15 min at room temperature. The cells were then blocked in 1× Incubation Buffer (10% Blocking Buffer with 0.1% Triton X-100 in PBS) for 15 min at room temperature. Then, cells were incubated with primary antibodies against the mitochondrial trifunctional protein subunit alpha (HADHA) at 1:50, or the IgG isotype control prepared in the 1× Incubation Buffer for 24 h at 4 °C. After washing, with a 1× Wash Buffer (0.05% Tween-20, 1% Blocking Buffer in PBS) the cells were incubated for 1 h in the dark at room temperature with a fluorochrome-conjugated goat anti-mouse secondary antibody (Alexa Fluor^®^ 488) at 1:100 dilution in the 1× Incubation Buffer. After final washes, cells were resuspended in PBS at 1 × 10^6^ cells/mL and transferred from Eppendorf tubes through a cell strainer and into 25 mL polystyrene tubes for flow cytometry analysis. Using the BD FACSCanto-II machine, fluorescence signals were collected for each sample and subsequently analyzed. To assess FAO activity across different cell lines, the percentage of cells expressing the HADHA protein was measured. Fluorescent intensity in the P2 and P3 gates, which are gated from the parent population, P1, was utilized as indicators of HADHA presence. The P1 gate identifies the total viable cell population, while the P2 and P3 gates identify the cell populations that express the HADHA protein. Comparisons were made between IgG controls and HADHA-stained samples in both untreated and PunA-treated groups. The difference in HADHA-positive cells between the treated and untreated conditions was used to calculate the FAO difference, representing relative changes in FAO activity. This experiment was independently performed two times.

### 2.6. Measurement of Oxygen Consumption Rate (OCR)

The OC cells HEYA8 and normal fallopian tube cells P201 were seeded into Seahorse XF cell culture microplates (Cat # 103793-100, Agilent, Santa Clara, CA, USA) at a density of 15 × 10^3^ cells/well and 20 × 10^3^ cells/well, respectively, and incubated overnight. Cells were then treated with either vehicle control (methanol) or PunA at concentrations of 20 μM, 40 μM, and 60 μM for 48 h. PunA was dissolved in methanol and further diluted in culture medium, and the vehicle control contained the same methanol concentration as the highest PunA condition. Then, the oxygen consumption rate (OCR) was measured using the XFe96 Extracellular Flux Analyzer (Seahorse Bioscience) at Duke Core Facility. One hour prior to measurement, the culture medium was replaced with Seahorse assay medium (Cat # 103680-100, Agilent, Santa Clara, CA, USA) supplemented with 10 mM glucose, 2 mM glutamine, and 1 mM sodium pyruvate (Cat # 103680-100, Agilent, Santa Clara, CA, USA). The following compounds were sequentially injected during the assay: 1.5 μM oligomycin, 1.5 μM FCCP, and 0.75 μM rotenone + 0.75 μM antimycin A (Cat # 103010-100, Agilent, Santa Clara, CA, USA). OCR was measured according to the XF Cell Mito Stress Test protocol from the manufacturer. The OCR measurements were normalized with the protein concentrations in each well of the cell culture plate which were determined using the Pierce™ BCA Protein Assay Kit (Cat # 23225; Thermo Fisher, Norristown, PA, USA), following the manufacturer’s instructions. Each treatment condition was tested in six technical replicates per experiment, and the experiment was performed in three biological replicates.

### 2.7. Peroxisome Proliferator-Activated Receptor Gamma (PPAR-γ) Assay

HEYA8 and P201 cells were seeded in 10 cm Petri-dishes at densities of 4 × 10^5^ cells/plate and 1 × 10^6^ cells/plate, respectively. After overnight incubation, cells were treated with either vehicle control (methanol) or various concentrations of PunA for 48 h. PunA was dissolved in methanol and further diluted in culture medium, and vehicle controls were prepared to match the highest methanol concentration corresponding to the maximum PunA dose. Following treatment, cells were scraped, and total cellular proteins were extracted for analysis. PPAR-γ protein levels were quantified using Human PPAR-gamma ELISA kit (Cat # MBS2503174, MyBioSource, San Diego, CA, USA) according to the manufacturer’s instructions. Total protein concentrations were measured using a BCA assay, and equal amounts of protein were used for subsequent PPAR-γ quantification. All measurements were performed in triplicate independent experiments. Each treatment condition was tested in two technical replicates per experiment, and the experiment was performed in three biological replicates.

### 2.8. Animal Ethics and Husbandry

All animal experiments were approved by the Duke University Institutional Animal Care and Use Committee (IACUC; Protocol # A223-21-11) and were conducted in accordance with the National Institutes of Health (NIH) Guide for the Care and Use of Laboratory Animals. Mice were housed under Specific Pathogen-Free (SPF) conditions in ventilated cages (up to five mice per cage) at a temperature of 22 ± 2 °C, a relative humidity of 40–60%, and a 12 h light/dark cycle. Mice had ad libitum access to standard chow and water. Following tumor cell injection, mice were monitored at least twice per week for 3–4 weeks or until predefined humane endpoints were reached. Humane endpoints included >20% body weight loss, development of ascites, tumor burden exceeding 10% of body weight, or obvious signs of distress. Mice that met these criteria were immediately humanely euthanized.

### 2.9. Tumor Development in C57BL/6J Mouse Model

An OC model was established in 10 female C57BL/6J mice, including five 8-week-old mice (young) purchased from Charles River and five 19-month-old mice (aged) obtained from the National Institute on Aging (NIA, Bethesda, MD, USA). Mice were allocated into experimental groups based on predefined age categories (young versus aged). As age was the primary biological variable under investigation, randomization was not applicable for age grouping. Body weight was recorded prior to tumor cell injection to ensure comparable baseline characteristics between groups.

Each mouse received a single intraperitoneal (IP) injection of 5 × 10^6^ ID8-Luc cells suspended in 200 µL of a 1:1 mixture of Matrigel and PBS. The intraperitoneal route was selected to mimic the peritoneal dissemination pattern observed in human OC. Tumor formation and growth were monitored by IVIS bioluminescence imaging on days 11 and 18 after injection. All mice were euthanized under IACUC-approved protocols using CO_2_ asphyxiation as the primary method followed by bilateral thoracotomy as the secondary method.

### 2.10. PunA Preventive Treatment Using C57BL/6J Mouse Model

Four female C57BL/6J mice (8 weeks old) were purchased from Charles River and housed under SPF conditions. All mice received a single intraperitoneal injection (IP) of 5 × 10^6^ ID8-Luc ovarian carcinoma cells suspended in 200 µL of a 1:1 mixture of Matrigel and PBS. Three days after tumor cell injection, mice were randomly assigned to control and treatment groups using a random number generator. One mouse was allocated to the vehicle control group, and the remaining three mice were treated with PunA. Due to the exploratory nature of this pilot study, a formal sample size calculation was not performed. Baseline body weights were recorded prior to randomization to ensure no systematic allocation bias.

PunA stock solution (10 mg/mL) was prepared in methanol and subsequently diluted in PBS immediately before administration to animals. Beginning three days after tumor cell injection, PunA was administered via IP injection once daily for 3 weeks at a dose of 30 µg/mouse in 100 µL total volume, resulting in a final methanol concentration of 3% (*v*/*v*). Control mice received vehicle diluted in PBS (100 µL of 3% (*v*/*v*) methanol), ensuring that the same final methanol concentration was applied to both control and treated groups. Tumor formation and growth were monitored weekly by IVIS bioluminescence imaging. After 3 weeks of treatment, all mice were euthanized under IACUC-approved protocols using CO_2_ asphyxiation as the primary method followed by bilateral thoracotomy as the secondary method.

### 2.11. IVIS Bioluminescence Imaging

Bioluminescence imaging was performed using the IVIS Spectrum system (PerkinElmer, Waltham, MA, USA). During imaging, mice were anesthetized and maintained with 2% isoflurane. D-luciferin potassium salt was administered via intraperitoneal injection at a dose of 150 mg/kg. Imaging was conducted 10–15 min post-injection. Total photon flux was quantified using Living Image software (version 4.3.1) with background correction.

### 2.12. RNA Extraction and RT-qPCR

Total RNA was extracted using RNA STAT60 Reagent^®^ (Cat # CS-111, Tel-Test, Friendswood, TX, USA). The RNA concentration was determined using Nano-drop2000. RT-qPCR was carried out using 300 ng of RNA per reaction from the methanol control and PunA-treated HEYA8 cells in a 20 μL reaction volume using the SuperScript IV One-Step RT-PCR kit (Cat # 12594025, Thermo Fisher, Norristown, PA, USA) according to the protocol provided by the manufacturer with Taqman probes for the genes of Solute Carrier Family 7 Member 11 (*SLC7A11*) and Glutathione-specific gamma-glutamylcyclotransferase 1 (*CHAC1*). A probe for the *GAPDH* gene was used in every RT-qPCR reaction as an internal control for each gene expression analysis. This experiment was independently performed two times.

### 2.13. RNA Extraction and RNA Sequencing

OC cell lines (HEYA8, A2780) and normal epithelial cells (P201, E7) were treated with PunA (15 μM for HEYA8 and A2780; 20 μM for P201 and E7) or vehicle control for 48 h. PunA was dissolved in methanol and diluted in culture medium, resulting in a final methanol concentration of approximately 0.04–0.06% (*v*/*v*) depending on the treatment condition. Vehicle controls were prepared to match the corresponding methanol concentration in PunA-treated samples. Each condition was analyzed with two technical replicates. Total RNA was extracted using RNA STAT60 Reagent^®^ (Cat # CS-111, Tel-Test, Friendswood, TX, USA). RNA libraries were prepared and sequenced by Admera Health (South Plainfield, NJ, USA) using an Illumina sequencing platform. Approximately 21–28 million reads were generated per sample.

### 2.14. RNA-seq Data Analysis

Raw sequencing reads were quality-checked using FastQC (v0.11.8) and summarized with MultiQC. Adapter trimming and removal of low-quality bases were performed using Trimmomatic (v0.38). Clean reads were aligned to the reference genome using STAR aligner (v2.7.1a), and duplicate reads were marked using Picard tools (v2.20.4). Alignment statistics indicated that 96–97% of reads mapped to the reference genome, with 93–95% uniquely mapped reads, indicating high sequencing and alignment quality.

Gene-level read counts were generated using HTSeq, and transcript assembly was performed using StringTie (v2.0.4). Differential gene expression analysis was conducted using DESeq2 (v1.14.1) based on raw count data. For baseline comparisons between cancer and normal cells, standard statistical criteria were applied, and *p*-values were adjusted using the Benjamini–Hochberg false discovery rate (FDR) method. For treatment-induced transcriptional changes, due to the use of technical replicates, statistical power was limited. Therefore, differential expression was primarily assessed based on fold change rather than statistical significance. Genes with an absolute log_2_ fold change ≥ 1 were selected for downstream exploratory analyses. Gene set enrichment analysis (GSEA) was performed using the clusterProfiler package within the Bioconductor framework (v3.20) in R (v4.4.2), based on ranked gene lists. KEGG pathway enrichment analysis was conducted as an exploratory approach to identify pathways associated with transcriptional changes.

### 2.15. Statistical Analysis

Cell viability was assessed using the MTS assay, comparing PunA treatment to methanol vehicle controls. Data are presented as mean ± standard deviation (SD). Statistical analyses were performed using GraphPad Prism (version 10.5.0). Differences between two groups were evaluated using unpaired Student’s *t*-tests. For comparisons involving a single factor with more than two groups, one-way ANOVA was performed. For experiments involving multiple variables, two-way ANOVA was used to evaluate main effects and interactions. Post hoc multiple comparisons were conducted using Dunnett’s, Tukey’s or Šídák’s test, as appropriate. Statistical significance was defined as *p* < 0.05.

## 3. Results

### 3.1. Aging Promotes Tumor Expansion in Immunocompetent Mice

**Figure 1:**
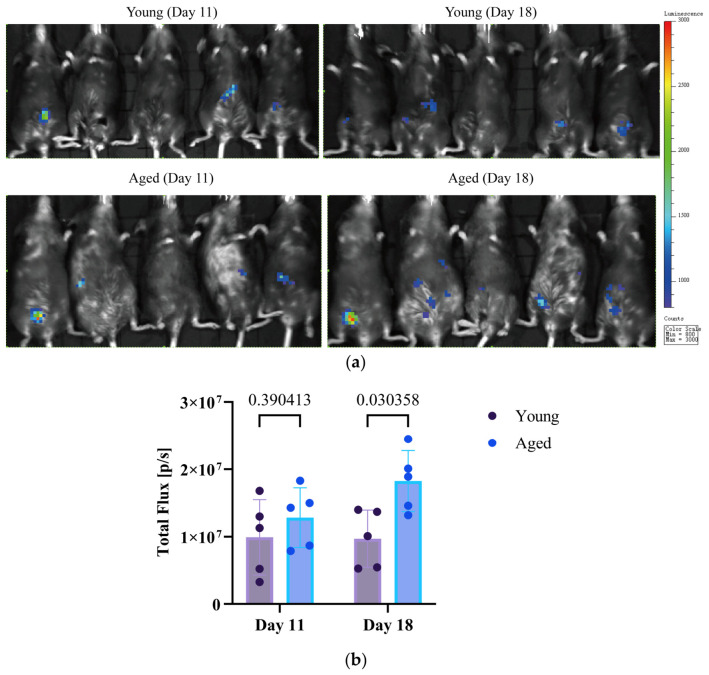
Aged mice demonstrated a larger tumor burden compared to young mice. (**a**) ID8-OC cells were intraperitoneally injected into young (8-week-old) and aged (19-month-old) female C57BL/6 mice. Tumor burden was assessed by in vivo bioluminescence imaging (IVIS). Representative IVIS images obtained on days 11 and 18 after tumor cell injection are shown. (**b**) The total flux was measured for young (8-week-old) and aged (19-month-old) female C57BL/6 mice. The mean values of total flux were used for the comparison of tumor formation on day 11 and day 18 after OC cell injection. Quantification of total photon flux (p/s) revealed a trend toward higher tumor-associated signal in aged mice compared with young mice at day 11 (*p* = 0.39), with the difference becoming statistically significant at day 18 (*p* = 0.03). Statistical analysis was performed using an unpaired two-tailed Student’s *t*-test, and data are presented as mean ± SD. *p* value < 0.05 was considered statistically significant.

Previously, we found that OC development is more pronounced in aged RNU athymic nude rats [13], whose lack of a functional thymus and T cells renders them severely immunodeficient in terms of cellular immunity. In the current study, we expanded upon these findings by examining OC progression in the C57BL/6 mouse model, a strain widely used in immunological research because of its robust immune response [26]. In this model, mouse ID8-OC cells were intraperitoneally inoculated into young (8 weeks) and aged (19 months) C57BL/6 mice. Bioluminescence imaging (IVIS) live imaging was performed on days 11 and 18 post-injection (Figure 1). Aged mice consistently exhibited stronger abdominal bioluminescence signals at both timepoints (Figure 1a). Quantitative IVIS measurements confirmed significantly higher total photon flux in aged mice compared to young controls at both 11 and 18 days post-injection (Figure 1b), indicating accelerated tumor growth and dissemination in older hosts like we have identified with the nude rats [13]. The longitudinal imaging demonstrated a progressive increase in tumor-associated photon flux across both cohorts. On day 11, aged mice showed a non-significant trend toward higher signal intensity relative to young mice (*p* = 0.39). By day 18, however, this difference reached statistical significance (*p* = 0.03), demonstrating that aging markedly enhances tumor expansion in this immunocompetent model.

### 3.2. PunA Reduces Tumor Burden in an ID8 Ovarian Cancer Mouse Model

**Figure 2:**
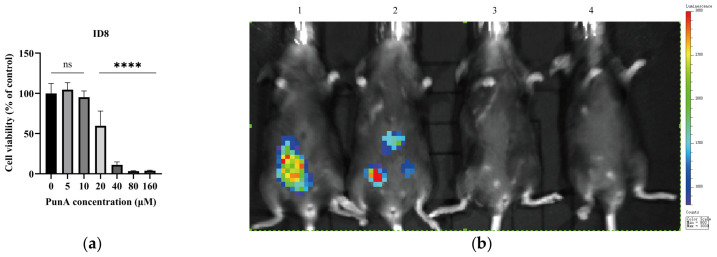
Effects of Punicic Acid on ID8 Ovarian Cancer Cells In Vitro and Tumor Growth In Vivo. (**a**) ID8 cells were treated with increasing concentrations of PunA (0–160 μM) for 48 h. PunA was dissolved in methanol and further diluted in culture medium before using, with methanol vehicle controls containing the same methanol concentration as the highest PunA treatment condition. Cell viability was assessed by MTS assay. PunA reduced cell viability in a dose-dependent manner, with significant inhibition observed at concentrations of ≥20 μM compared with control. Data are presented as mean ± SD of six technical replicates, representative of three independent experiments; Statistical analysis was performed using one-way ANOVA followed by Dunnett’s multiple comparisons test. ns, not significant; **** *p* < 0.0001. (**b**) Young (8-week-old) C57BL/6 mice were intraperitoneally injected with ID8-OC cells. Starting on day 3 post-injection, mice received daily intraperitoneal administration of PunA (30 μg/mouse) for 3 weeks. In vivo bioluminescence imaging (IVIS) was performed at the end of week 3 of treatment to assess tumor burden. Representative IVIS images are shown, with mouse #1 representing the control group and mice #2–4 representing the PunA-treated group.

In a previous study, we examined the dose-dependent cytotoxicity of PunA using OC cell lines A2780, HEYA8, and CAOV2, which suggested that PunA may possess therapeutic potential in cancer prevention and treatment [13]. Building on these findings, we next evaluated PunA in an ID8-OC syngeneic model using immunocompetent C57BL/6 mice. The effects of PunA on ID8-OC cells were first assessed in vitro using concentrations ranging from 5 to 160 µM (Figure 2a). PunA was dissolved in methanol (vehicle controls) and further diluted in culture medium before using, with vehicle controls matched to the highest methanol concentration. PunA reduced cell viability in a dose-dependent manner, with significant effects observed at concentrations ≥ 20 uM (*p* < 0.05). To explore its potential in vivo effects, young C57BL/6 mice were intraperitoneally injected with ID8-OC cells and, beginning on day 3 post-injection, treated daily with PunA (30 μg/mouse) for 3 weeks. Bioluminescence imaging at the end of treatment revealed that PunA-treated mice (*n* = 3) tended to exhibit lower tumor-associated photon flux compared to the untreated control (*n* = 1) (Figure 2b). Although these data suggest that PunA may reduce tumor burden in vivo, interpretation is limited by the small sample size and the lack of statistical analysis, primarily due to the use of only a single control mouse. Thus, validation in larger cohorts is needed to confirm the in vivo therapeutic potential of PunA.

### 3.3. PunA Preferentially Reduces Ovarian Cancer Cell Viability

**Figure 3:**
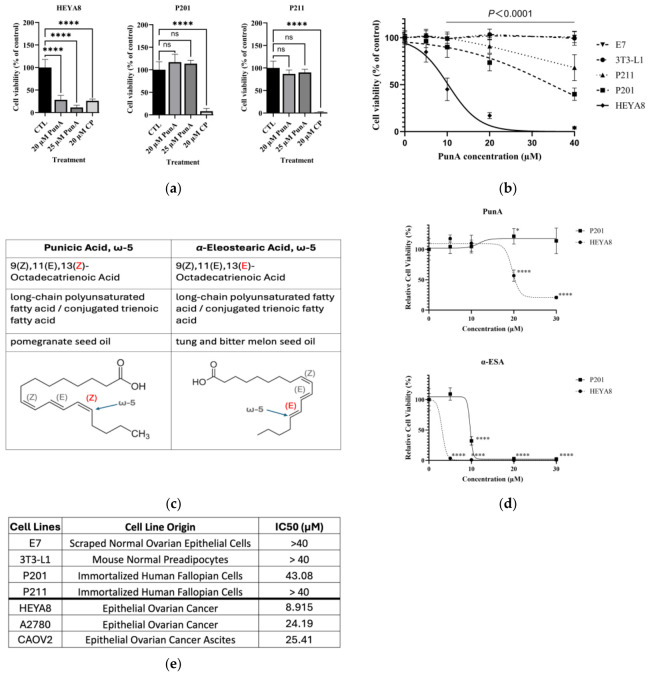
Differential Effects of Punicic Acid and α-Eleostearic Acid on OC and Normal Cell Lines. (**a**) HEYA8 OC cells and NFT epithelial cell lines (P201 and P211) were treated for 48 h with methanol control (CTL, black bars), 20 µM PunA (medium grey bars), 25 µM PunA (dark grey bars) or 20 µM cisplatin (CP, light grey bars). Data are presented as mean ± SD of six technical replicates, representative of three independent experiments. Statistical significance was determined by one-way ANOVA with Dunnett’s multiple comparisons test versus control. **** *p* < 0.0001, ns: not significant. (**b**) OC cells and normal cell lines were treated with increasing concentrations of PunA (0, 5, 10, 20, and 40 μM) for 48 h, and cell viability was determined by the MTS assay. Data represent mean ± SD from three independent experiments. Statistical analysis was performed using two-way ANOVA followed by Dunnett’s multiple comparisons test. No significant differences were observed at baseline (0 μM; all *p* > 0.9999). At 5 μM, HEYA8 cells showed significantly lower viability compared to P201 (*p* = 0.0196), P211 (*p* = 0.0002), E7 (*p* = 0.0005), and 3T3-L1 (*p* = 0.0002), with all comparisons reaching *p* < 0.0001 at higher concentrations. (**c**) Structural comparison of PunA (cis-9, trans-11, cis-13) and α-ESA (cis-9, trans-11, trans-13), both conjugated trienoic FFAs differing only in the π-bond orientation at the omega-5 carbon. (**d**) HEYA8 and P201 cells were treated for 48 h with varying concentrations of PunA and α-ESA. While α-ESA reduced viability in both HEYA8 and P201 cells, PunA showed a dose-dependent reduction in HEYA8 viability with minimal cytotoxicity in P201 cells. Data are presented as mean ± SD of six technical replicates, representative of three independent experiments. Statistical significance was determined by two-way ANOVA with Dunnett’s post hoc test versus control. * *p* < 0.05, **** *p* < 0.0001. (**e**) Summary table of PunA IC_50_ values between normal cell lines (top four rows) and OC cell lines (bottom three rows).

Cisplatin has long been a first-line chemotherapeutic for OC patients, but its clinical use in long term treatment is limited by its toxicity to normal tissues [27]. To compare the cytotoxicity of PunA with cisplatin, HEYA8 OC cells and two immortalized NFT epithelial cell lines (P201 and P211) were treated for 48 h with vehicle control (methanol), 20 µM PunA, 25 µM PunA, or 20 µM cisplatin (Figure 3a). MTS assay results revealed that PunA preferentially reduced HEYA8 viability by >70% at both doses, while P201 and P211 viabilities were largely preserved. In contrast, cisplatin reduced viability by >70% in HEYA8 and caused even greater reductions in P201 and P211, confirming its non-selective cytotoxicity.

To further assess cancer selectivity, HEYA8 and four normal cells lines—P201, P211, mouse preadipocytes (3T3-L1) and normal ovarian epithelial cells (E7)—were exposed to increasing concentrations of PunA for 48 h (Figure 3b). Two-way ANOVA revealed significant effects of PunA concentration (F(4125) = 142.0, *p* < 0.0001), cell line (F(4125) = 266.5, *p* < 0.0001), and a significant interaction between concentration and cell line (F(16,125) = 40.82, *p* < 0.0001), indicating that the response to PunA differed among cell lines. Post hoc analysis using Dunnett’s multiple comparisons test showed no significant differences between HEYA8 and non-tumor cell lines (P201, P211, E7, and 3T3-L1) at baseline (0 μM; all adjusted *p* > 0.9999). However, at 5 μM, HEYA8 cells exhibited significantly reduced viability compared to P201 (*p* = 0.0196), P211 (*p* = 0.0002), E7 (*p* = 0.0005), and 3T3-L1 (*p* = 0.0002). At higher concentrations (≥10 μM), these differences became highly significant across all comparisons (all *p* < 0.0001), with progressively larger reductions in viability observed in HEYA8 cells relative to non-tumor cell lines.

Another member of omega-5 FFA 18:3 family, α-ESA, has very high structural similarity to PunA and is categorized as its geometric isomer (Figure 3c). To determine whether PunA’s effects extend to α-ESA, HEYA8 and P201 cells were treated with increasing concentrations of each compound for 48 h (Figure 3d). MTS assay results demonstrated that α-ESA reduced viability in both cancerous and normal cells, indicating non-selective toxicity. In contrast, PunA decreased HEYA8 viability in a dose-dependent manner while minimally affecting P201 cells. Summarized IC_50_ values for PunA (Figure 3e) further quantify this selectivity: normal cell lines consistently exhibited higher IC_50_ values (>40 µM), whereas OC cell lines displayed much lower IC_50_ values (~25 µM or even lower). The highly sensitive response of HEYA8 cells to PunA (IC_50_ = 8.915), is significant, as HEYA8 cells are derived from high-grade serous ovarian cancer (HGSOC), the most prevalent and aggressive epithelial OC histological subtype [28]. Additionally, CAOV2 cells exhibited a relatively sensitive response to PunA (IC_50_ = 25.41) compared to normal cell lines. This is notable given we previously found that CAOV2 cells, derived from OC ascites, exhibit chemoresistance to conventional chemotherapies under acidic conditions characteristic of the ascites TME [29]. Collectively, these results suggest that PunA preferentially reduces OC cell viability compared with the tested normal cell lines, distinguishing it from α-ESA.

### 3.4. Cooperative Potential Between PunA and Cisplatin

**Figure 4:**
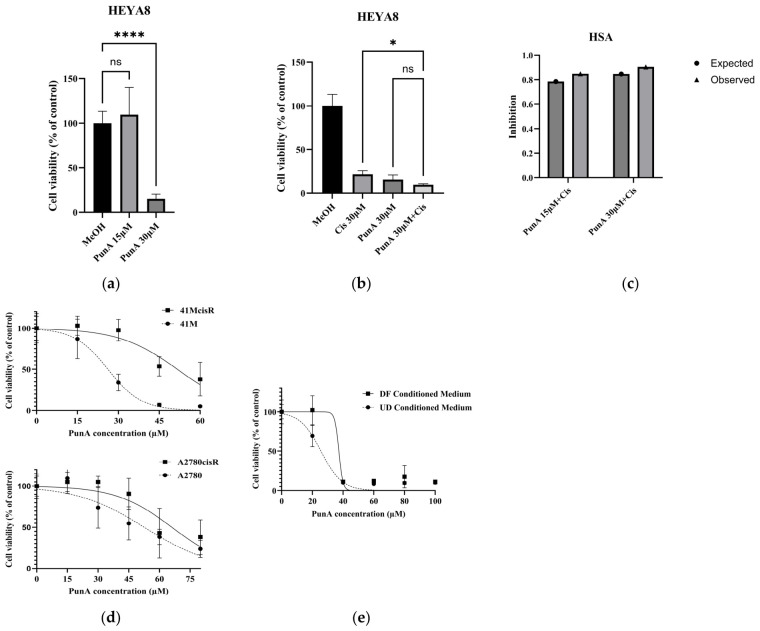
Effects of PunA Alone and in Combination with Cisplatin on Cell Viability, Drug Interaction, Resistance, and Lipidic Microenvironment. (**a**) HEYA8 cells were treated with PunA (15 or 30 μM) for 48 h, and cell viability was measured using an MTS assay. Statistical significance was determined by one-way ANOVA with Dunnett’s multiple comparisons test versus control. (**b**) HEYA8 cells were treated with cisplatin (30 μM), PunA (30 μM), or their combination for 48 h, followed by MTS analysis. Statistical significance was determined using one-way ANOVA followed by Šídák’s multiple comparisons test. (**c**) Drug interaction between PunA and cisplatin was evaluated using the Highest Single Agent (HSA) model by comparing predicted and observed inhibition. Data (**a**–**c**) are presented as mean ± SD of six technical replicates, representative of two independent experiments. * *p* < 0.05, **** *p* < 0.0001, ns: not significant. (**d**) Cisplatin-sensitive and -resistant cell lines (41M/41McisR and A2780/A2780cisR) were treated with increasing concentrations of PunA for 48 h (0 μM–60 μM for 41M pair, upper panel; 0 μM–75 μM for A2780 pair, lower panel). Data are presented as mean ± SD of six technical replicates, representative of three independent experiments. Statistical significance was assessed by two-way ANOVA with Dunnett’s post hoc test. For 41M/41McisR, the interaction, PunA factor, and drug-resistance factor were all significant (*p* < 0.0001). For A2780/A2780cisR, the interaction was significant (*p* = 0.0341), as were the PunA factor (*p* < 0.0001) and drug-resistant factor (*p* = 0.0019). (**e**) HEYA8 cells were cultured in DF or UD 3T3-L1 conditioned medium and treated with increasing concentrations of PunA (0–100 μM) for 48 h. Cell viability was measured by MTS assay, normalized to untreated controls. Data are presented as mean ± SD of six technical replicates, representative of three independent experiments. Statistical significance was assessed by two-way ANOVA with Dunnett’s post hoc test. The interaction was significant (*p* = 0.0003), along with the PunA factor (*p* < 0.001) and DF conditioned medium factor (*p* = 0.0008).

To assess the cytotoxic effects of PunA alone and in combination with cisplatin, HEYA8 cells were treated with PunA (15 or 30 μM), cisplatin (30 μM), or their combination for 48 h, and cell viability was measured using an MTS assay. Consistent with our earlier findings, PunA reduced HEYA8 cell viability in a dose-dependent manner, with 30 μM producing a significant inhibitory effect, whereas 15 μM showed no significant effect under these conditions (Figure 4a). We next compared the effects of single-agent and combination treatments. Cisplatin alone significantly reduced cell viability relative to the control, and PunA alone (30 μM) also showed a marked inhibitory effect (Figure 4b). Notably, the combination of PunA and cisplatin further decreased cell viability compared with cisplatin treatment alone. However, no significant difference was observed between the PunA single-treatment group and the combination group. To further evaluate the interaction between PunA and cisplatin, drug interaction was analyzed using the Highest Single Agent (HSA) model (Figure 4c). The observed inhibitory effect of the combination treatment was slightly greater than the predicted inhibition based on the most active single agent, suggesting a potential cooperative interaction between PunA and cisplatin under these experimental conditions.

### 3.5. Chemo-Resistant OC Cells Exhibit Diminished Sensitivity to PunA

An additional challenge in OC treatment is the frequent emergence of chemoresistance, especially when long-term chemotherapy is applied [30,31]. To evaluate the effects of PunA in cisplatin-resistant OC cells, cisplatin-sensitive and resistant lines (41M/41McisR and A2780/A2780cisR) were treated with PunA at concentrations ranging from 0 μM to 75 μM increasing in 15 μM increments (Figure 4d). The data showed that PunA reduced cell viability in a dose-dependent manner; however, this efficacy was diminished in the cisplatin-resistant cells relative to their parental counterparts in both cell pairs. Our study showed that PunA did not display enhanced activity against cisplatin-resistant cells, suggesting that resistance mechanisms limit cancer cell response to PunA.

### 3.6. PunA Remains Effective in High-Fat Environments

Next, we tested the sensitivity of PunA in OC cells cultured under a high-fat environment (Figure 4e), a common condition in both micro- and macro-environments in patients with OC [32]. 3T3-L1 preadipocytes were differentiated into adipocytes using a standard hormone cocktail, including insulin, which led to the accumulation of lipid droplets. This process is a well-established model for studying fat cell biology and adipogenesis [33]. Conditioned medium was collected from DF and UD 3T3-L1 cultures and used to mimic fatty versus non-fatty conditions, respectively.

HEYA8 cells were cultured in DF or UD 3T3-L1 conditioned medium and treated with increasing concentrations of PunA for 48 h. PunA reduced cell viability in a dose-dependent manner under both conditions. Cells cultured in UD conditioned medium exhibited greater sensitivity to PunA, showing a more pronounced decrease in viability at lower concentrations compared to those cultured in DF conditioned medium. Two-way ANOVA revealed significant effects of PunA treatment (*p* < 0.001), conditioned medium (*p* = 0.0008), and their interaction (*p* = 0.0003), indicating that the response to PunA is significantly influenced by the metabolic environment.

### 3.7. PunA May Promote Ferroptosis in Ovarian Cancer Cells

**Figure 5:**
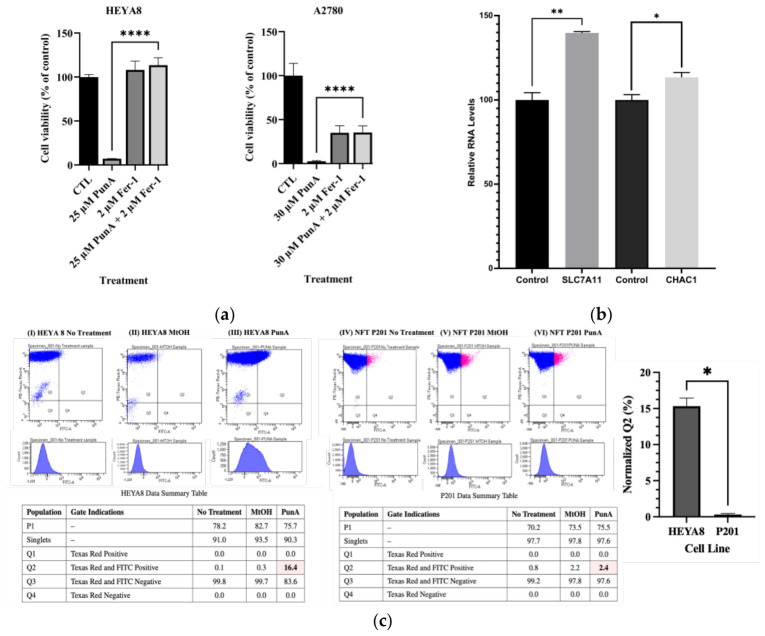
PunA induces OC cell ferroptosis and lipid peroxidation. (**a**) HEYA8 cells and A2780 cells were treated with PunA (25 μM for HEYA8 or 30 μM for A2780), 2 μM of Fer-1, and the combination of Fer-1 and PunA for 48 h. The cell viability was tested using MTS assay. Data is presented as mean ± SD of six technical replicates, representative of four independent experiments. Statistical significance was determined by one-way ANOVA with Tukey’s post hoc test. **** *p* < 0.0001. (**b**) HEYA8 cells were treated with 25 μM PunA or mock treatment control (methanol) for 48 h. Expression of *SLC7A11* and *CHAC1* was measured by RT-qPCR. Data are presented as mean ± SD of two independent experiments. Statistical significance was assessed using an unpaired *t*-test. *SLC7A11* expression was significantly altered (*p* = 0.0061), as was *CHAC1* (*p* = 0.0467). * *p* < 0.05, ** *p* < 0.01. (**c**) BODIPY 581/591 C11 staining and flow cytometry analysis were performed on HEYA8 and P201 cells after the cells were untreated (left panel), treated with methanol (MtOH, middle panel), or 10 μM of PunA (right panel) for 24 h. Q1 showed the percentage of cells that were Texas Red single positive, Q2 was Texas Red and FITC double positive, Q3 was Texas Red and FITC double negative, and Q4 was FITC single positive for each of the treatment types. HEYA8 cells show a noticeable shift from Q1 to Q2 compared to the control and methanol vehicle control groups while P201 cells show a negligible shift, indicating a greater percentage of HEYA8 cells undergoing PunA-associated lipid peroxidation. Percentage of Q2-positive (Texas Red and FITC double-positive) cells, reflecting oxidized lipid accumulation measured by BODIPY 581/591 C11 staining. Percentage values represent PunA-treated cells after subtracting their respective methanol controls to account for methanol vehicle control. Data are presented as mean ± SD of two independent experiments. Statistical significance was assessed using an unpaired *t*-test (*p* = 0.0029). ** *p* < 0.01.

Many lipid-derived factors, particularly FFAs, exert cytotoxic effects by inducing ferroptosis, an iron-dependent, non-apoptotic form of cell death triggered by lipid peroxidation [34]. To determine whether ferroptosis mediates PunA-induced cell death, HEYA8 and A2780 cells were co-treated with PunA and 2 µM Ferrostatin-1 (Fer-1), a potent, synthetic inhibitor of ferroptosis (Figure 5a). PunA alone reduced viability by 93% in HEYA8 cells and 97% in A2780 cells, whereas co-treatment with Fer-1 significantly rescued viability (*p* < 0.001). To further validate this mechanism, RT-qPCR was performed in HEYA8 cells treated with 25 µM PunA for 48 h (Figure 5b). PunA significantly increased the mRNA expression of ferroptosis-related genes. *SLC7A11* (*p* = 0.003) and *CHAC1* (*p* = 0.023) compared with the vehicle control (methanol) by 40% and by 15%, respectively. Taken together, these data suggest that ferroptosis likely contributes to PunA-induced OC cell death.

### 3.8. PunA Selectively Increases Lipid Peroxidation in OC Cells

Ferroptosis and lipid peroxidation are tightly linked, with iron-dependent lipid peroxidation serving as a key driver ferroptosis [35,36]. To determine whether PunA selectively induces lipid peroxidation in OC versus NFT cells, HEYA8 and P201 cells were treated with vehicle control (methanol) or 10 μM PunA for 24 h, followed by BODIPY 581/591 C11 staining and flow cytometry (Figure 5c). The Q2 population, representing the double-positive for both Texas Red and FITC, reflects cells undergoing lipid peroxidation.

Methanol controls produced minimal shifts in fluorescence, confirming low baseline oxidation stress. In contrast, PunA treatment dramatically increased lipid peroxidation in HEYA8 cells (16.4% Q-positive), while only a minimal increase was observed in P201 cells (2.4%). After normalization to their respective methanol controls, PunA treatment resulted in a net 16.1% increase in lipid peroxidation in HEYA8 cells versus only 0.2% in the P201 cells (Figure 5c). These results demonstrate that PunA selectively induces lipid peroxidation in OC cells but not in normal cells, supporting a model in which PunA selectively triggers ferroptosis through lipid peroxide accumulation in cancer cells.

### 3.9. PunA Selectively Upregulates PPAR-γ Activity in OC Cells

**Figure 6:**
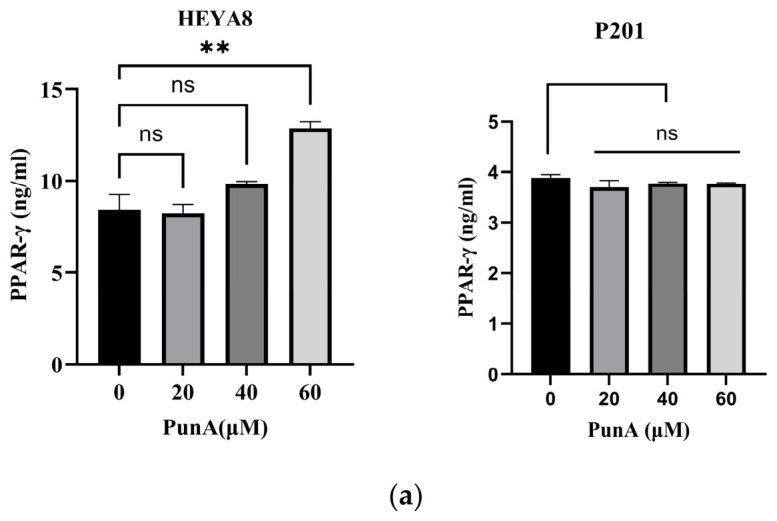

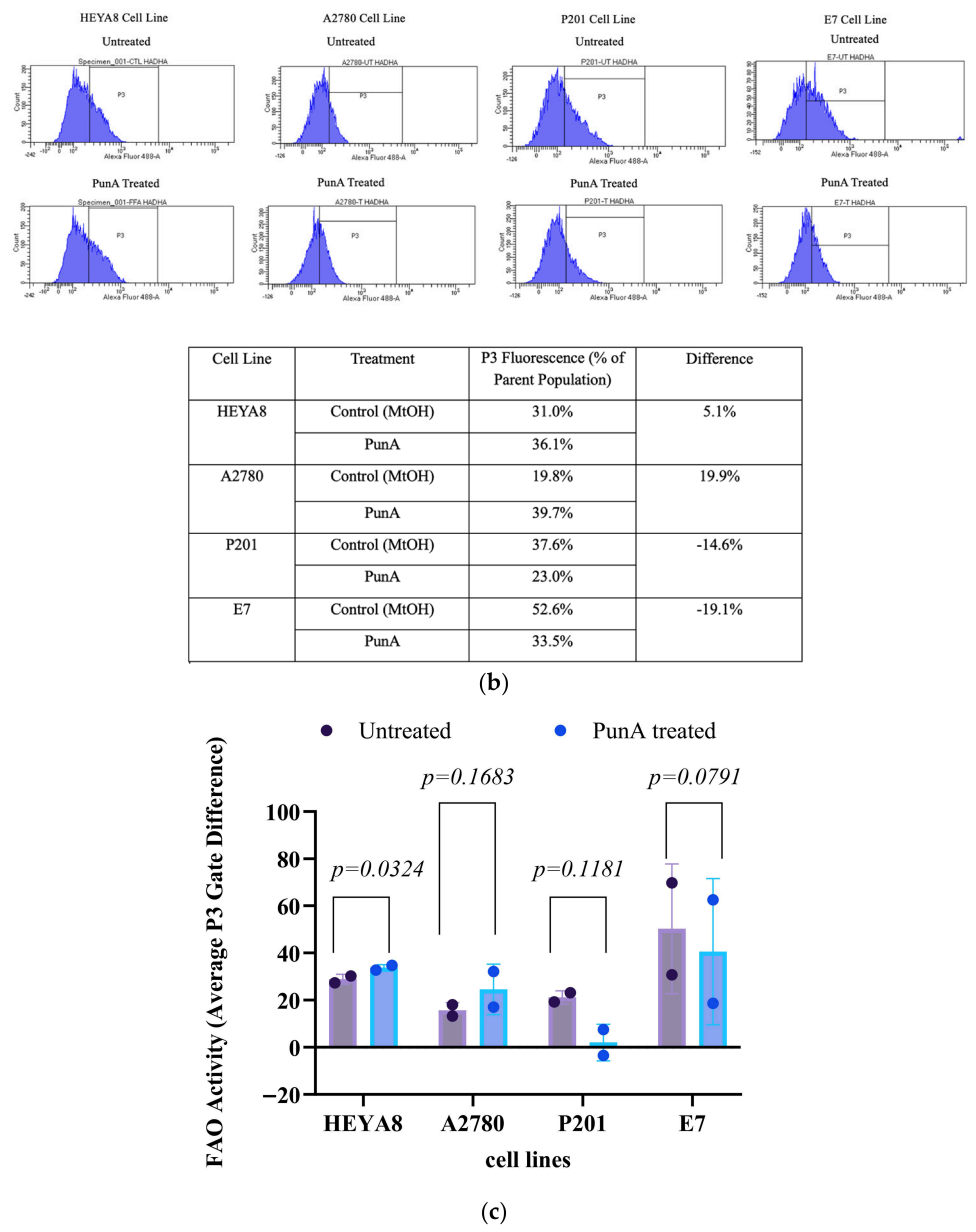
PPAR gamma and Fatty Acid Oxidation showed differential activities in cancer cells and noncancerous cells. (**a**) PunA induced PPAR-γ activity in HEYA8 at 60 μM (*p* = 0.0023) but not in P201 cells. HEYA8 (left) and P201 (right) cells were treated with increasing concentrations of PunA (0, 20, 40, and 60 μM) for 24 h. PPAR-γ activities were measured using an ELISA kit. Data is presented as mean ± SD from three independent experiments. Statistical analysis was performed using one-way ANOVA with Dunnett’s post hoc test. ** *p* < 0.01; ns: not significant. (**b**) Fatty Acid Oxidation (FAO) activity levels were examined by HADHA expression in cancer cell lines, HEYA8 and A2780, and noncancerous cell lines, P201 and E7, following PunA treatment. Flow cytometry analysis was used to analyze the HADHA expression which is shown in gate P3. This initial representation (without IgG control comparison) demonstrates the difference between the control (MtOH) and PunA treatment groups. Fluorescence in the P3 gate increased in cancerous cell lines and decreased in noncancerous cell lines between control and treatment groups. (**c**) Average FAO activity from two independent tests showed HADHA expression increasing in HEYA8 and A2780 cell lines with 10 μM PunA and 25 μM PunA respectively, compared to untreated groups. Average HADHA expressions decreased in noncancerous cell lines, P201 and E7, with 25 μM of PunA treatment compared to untreated groups. Statistical analysis was performed using a two-tailed paired student *t*-test with *p* < 0.05 considered significant for HADHA expressions (population percentage of gate 3).

Given the differential sensitivity of OC and NFT cells to PunA, we next examined the expression of Peroxisome Proliferator-Activated Receptor gamma (PPAR-γ) in HEYA8 and P201 cells (Figure 6a). PPAR-γ is a transcription factor that plays a central role in lipid metabolism, cell proliferation, and carcinogenesis [37]. PunA significantly upregulated PPAR-γ expression in HEYA8 cells in a dose-dependent manner, with a significant increase observed at 60 μM PunA (*p* = 0.0023). In contrast, PunA did not induce significant changes in PPAR-γ levels in P201 cells at any concentration, suggesting a cell-type-specific regulation of PPAR-γ by PunA.

### 3.10. PunA Induces Differential Fatty Acid Oxidation Activity

Fatty acid oxidation (FAO) is the primary metabolic pathway for generating cellular energy from fatty acids, a process that occurs primarily in the mitochondria [38]. FAO plays a multifaceted role in cancer by supplying energy that supports tumor growth, metastasis, stemness, and drug resistance in both cancer cells and the tumor microenvironment [38]. The hydroxyacyl-CoA dehydrogenase trifunctional multienzyme complex subunit alpha (HADHA) gene encodes a key mitochondrial enzyme complex that directly regulates the rate of FAO [39].

To assess how PunA affects FAO in PunA-treated OC and NFT cells, we first verified whether the FAO-related genes included in the assay kit (ACADVL, ACADM, and HADHA) were expressed in the OC cells HEYA8 and A2780. Flow cytometry analysis revealed detectable expression of only HADHA. After confirming HADHA expression in OC cells, we next quantified its expression following PunA treatment in four cell lines: HEYA8, A2780, P201, and E7. Flow cytometry analysis revealed distinct differences in HADHA expression between the OC cell lines (HEYA8 and A2780) and noncancerous epithelial cell lines (P201 and E7), as determined by P3 gating (Figure 6b). Data from two independent experiments are summarized in Figure 6c.

In both OC cell lines, PunA treatment increased HADHA expression, as evidenced by a higher percentage of cells within the P3 gate after correction for IgG control background. In HEYA8 cells, the proportion of cells in the P3 region increased significantly from an average of 28.85% in untreated cells to 33.75% following treatment with 10 μM of PunA (*p* = 0.03). A similar upward trend was observed in A2780 cells, where the proportion of cells in the P3 region rose from 15.65% in untreated cells to 24.55% after 25 μM PunA treatment. Although the increase in A2780 cells did not reach statistical significance (*p* = 0.17), the consistent elevation across both experiments supports a PunA-induced upregulation of HADHA in OC cells. In contrast, treatment of the noncancerous cell lines decreased the proportion of P3 cells, indicating reduced HADHA expression and, consequently, diminished FAO activity. In P201 cells, the P3 proportion declined from 21.15% in untreated cells to 2.00% after treatment with 25 μM PunA (*p* = 0.12), while E7 cells showed a reduction from 50.20% to 40.55% under the same conditions (*p* = 0.08). Although these changes did not reach statistical significance in every cell line, the consistent trend across experiments supports the hypothesis that PunA stimulates FAO in OC cells while repressing it in noncancerous epithelial cells.

### 3.11. PunA Selectively Impairs Mitochondrial Respiration in OC Cells

**Figure 7:**
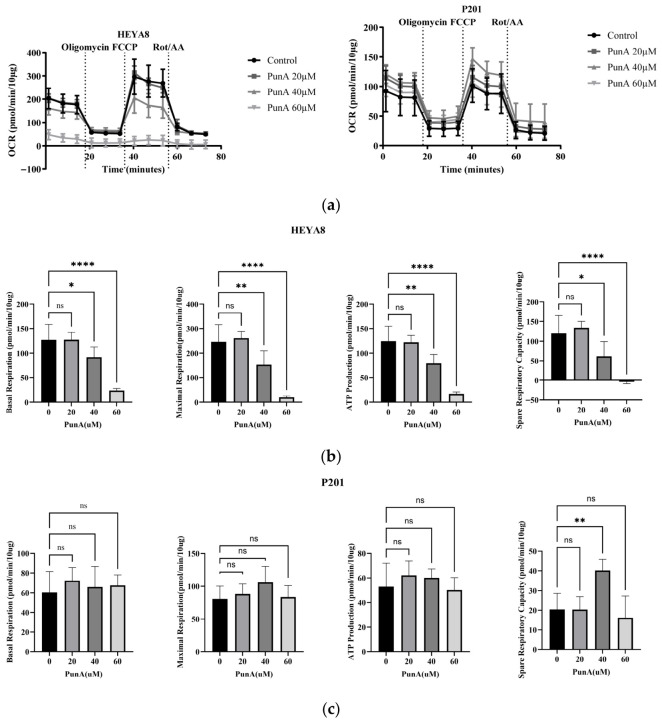
Differential effects of PunA on mitochondrial respiration in OC versus normal cells. (**a**) Oxygen consumption rate (OCR) profiles of OC HEYA8 (left) and NFT P201 (right) cells treated with different concentrations of PunA (20, 40, and 60 μM), assessed by Seahorse XF Mito Stress Test. Oligomycin, carbonyl cyanide-4-(trifluoromethoxy)phenylhydrazone (FCCP), and Rotenone/Antimycin A (Rot/AA) were sequentially injected at indicated time points. (**b**,**c**) Quantification of basal respiration, maximal respiration, ATP production, and spare respiratory capacity in HEYA8 (**b**) and P201 (**c**) cells. Data are presented as mean ± SD of six technical replicates, representative of three biological replicates. Statistical significance was determined by one-way ANOVA with Tukey’s post hoc test. * *p* < 0.05, ** *p* < 0.01, **** *p* < 0.0001, ns: not significant.

Given the importance of mitochondria in regulating lipid metabolism, we next assessed mitochondrial function using the Seahorse XF Cell Mito Stress Test. Treatment with PunA significantly suppressed mitochondrial respiration in HEYA8 cells in a dose-dependent manner (Figure 7a). As shown in the mitochondrial stress test, PunA notably reduced oxygen consumption rate (OCR) at all stages, particularly at higher concentrations (40 and 60 uM), indicating impaired mitochondrial function. Quantification of key parameters revealed marked decreases in basal respiration, maximal respiration, ATP production, and spare respiratory capacity in HEYA8 cells, with the most significant reductions observed at 60 uM PunA (Figure 7b). In contrast, P201 cells exhibited minimal or no significant changes in these parameters across all treatment groups (Figure 7c), suggesting that PunA selectively impairs mitochondrial respiration in tumor cells but not in normal fallopian tube epithelial cells.

### 3.12. PunA Induces a Stronger Oxidative Stress–Associated Transcriptional Response in Cancer Cells

**Figure 8:**
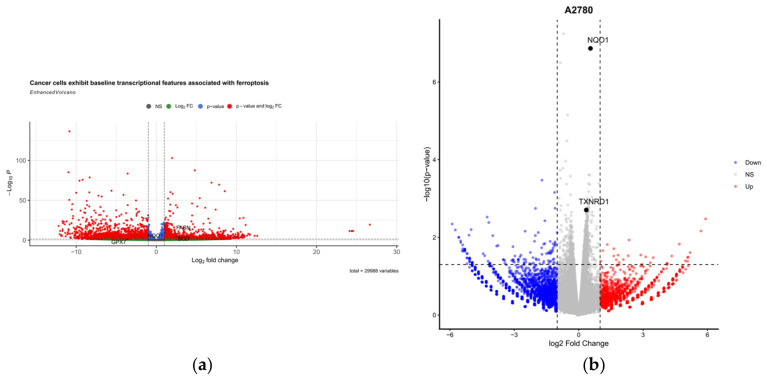

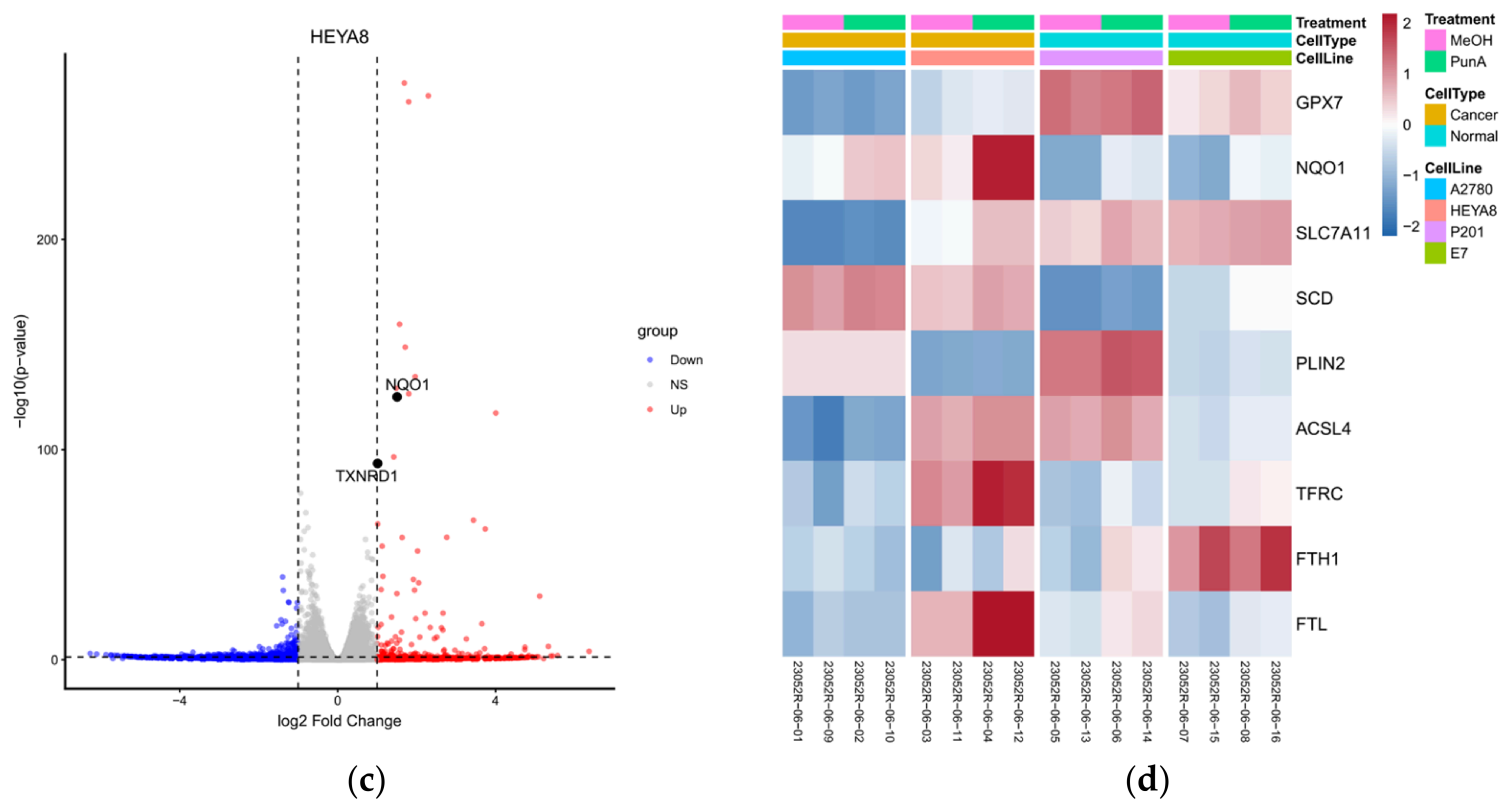
PunA induces ferroptosis-related transcriptional changes in cancer cells. (**a**) Volcano plot showing differential gene expression between cancer cell lines (A2780 and HEYA8) and normal epithelial cell lines (P201 and E7). Each point represents one gene, plotted by log_2_ fold change and −log_10_ *p*-value. Differential expression was assessed using standard statistical criteria and is shown here to illustrate global transcriptional differences between the two groups. Selected ferroptosis-related genes, including *NQO1*, *SCD*, *FASN*, and *GPX7*, are highlighted, indicating differences in pathways related to redox regulation and lipid metabolism. These baseline patterns suggest that cancer cells exhibit transcriptional features associated with ferroptosis-related processes. (**b**,**c**) Volcano plots showing transcriptional changes induced by PunA treatment in A2780 (**b**) and HEYA8 (**c**) cells. Each point represents one gene, plotted by log_2_ fold change and −log_10_ *p*-value. Due to the use of technical replicates, differential expression was primarily interpreted based on fold change (|log_2_ fold change| > 1) rather than strict statistical thresholds. Genes with increased expression are shown in red, and those with decreased expression are shown in blue. Representative redox-related genes, including *NQO1* and *TXNRD1*, are highlighted, illustrating consistent transcriptional responses associated with oxidative stress regulation across both cancer cell lines. (**d**) Heatmap showing expression patterns of ferroptosis-related genes across all samples. Rows represent selected genes associated with ferroptosis-related processes, including redox regulation, lipid metabolism, and iron homeostasis (e.g., *GPX7*, *NQO1*, *SLC7A11*, *ACSL4*, *TFRC*, *FTL*, and *FTH1*). Columns represent individual samples grouped by cell line, cell type, and treatment condition. The color scale indicates relative expression levels, with red representing higher expression and blue representing lower expression. Distinct expression patterns are observed between cancer and normal cells, as well as between PunA-treated and control samples, reflecting coordinated transcriptional variation in ferroptosis-related pathways.

To investigate PunA-induced transcriptional changes, RNA-seq was conducted on OC cells (HEYA8 and A2780) and normal epithelial cells (P201 and E7), under both PunA-treated and control (methanol) conditions. A total of 31,298 genes were analyzed across all samples following preprocessing and filtering steps (Table S2), with sample characteristics summarized in Table S1. Comparative analysis revealed clear transcriptional differences between cancer and normal cells at baseline (Figure 8a), including differential expression of genes associated with redox regulation and lipid metabolism. A comprehensive list of differentially expressed genes between cancer and normal samples is provided in Table S3, including a subset of ferroptosis-related genes (Table S4).

Upon PunA treatment, cancer cells exhibited pronounced transcriptional responses. In particular, oxidative stress–related genes, including *NQO1* and *TXNRD1*, were consistently upregulated (Figure 8b,c). The magnitude of induction differed between cell lines, with A2780 cells showing moderate changes (e.g., *NQO1* log2FC ≈ +0.55; *TXNRD1* ≈ +0.36), whereas HEYA8 cells exhibited markedly stronger upregulation (*NQO1* ≈ +1.50; *TXNRD1* ≈ +1.01), indicating enhanced sensitivity to PunA-induced redox perturbation. Detailed differential expression results for A2780 and HEYA8 cells are provided in Table S5 and Table S6, respectively, while expression changes in representative oxidative stress–related genes across cell lines are summarized in Table S7.

Consistent with these observations, HEYA8 cells displayed a broader transcriptional response compared to A2780 cells, suggesting a more pronounced cellular reaction to treatment. In contrast, normal cell lines (P201 and E7) exhibited relatively limited transcriptional changes following PunA treatment, as shown in Supplementary Figures S1 and S2. Key oxidative stress–related genes displayed minimal induction, and overall fewer genes exhibited substantial fold changes, indicating that normal cells maintain redox homeostasis under these conditions.

To further quantify these differences, we compared the expression change (Δ = PunA − MeOH) across cell types (Supplementary Figure S3). Cancer cells exhibited a consistently greater magnitude of transcriptional response than normal cells. However, gene-specific variability was observed; while most genes showed stronger induction in cancer cells, certain genes such as *PLIN2* displayed comparable or even higher changes in normal cells. Expression patterns of selected ferroptosis-related genes across all samples are shown in Figure 8d and Supplementary Figure S4. Together, these results demonstrate that PunA elicits a stronger but heterogeneous transcriptional response in cancer cells.

### 3.13. PunA Activates Ferroptosis-Associated Transcriptional Programs in Cancer Cells

To further characterize the biological processes underlying the observed transcriptional changes, we examined genes associated with ferroptosis. In cancer cells, PunA treatment induced coordinated upregulation of genes involved in oxidative stress (*NQO1*), lipid metabolism (*SCD*, *PLIN2*), and iron handling (*FTL*), representing key components of ferroptosis (Figure 8d). In contrast, normal cells exhibited weaker and less consistent transcriptional responses across these pathways, supporting a cancer-specific activation of ferroptosis-associated programs.

**Figure 9:**
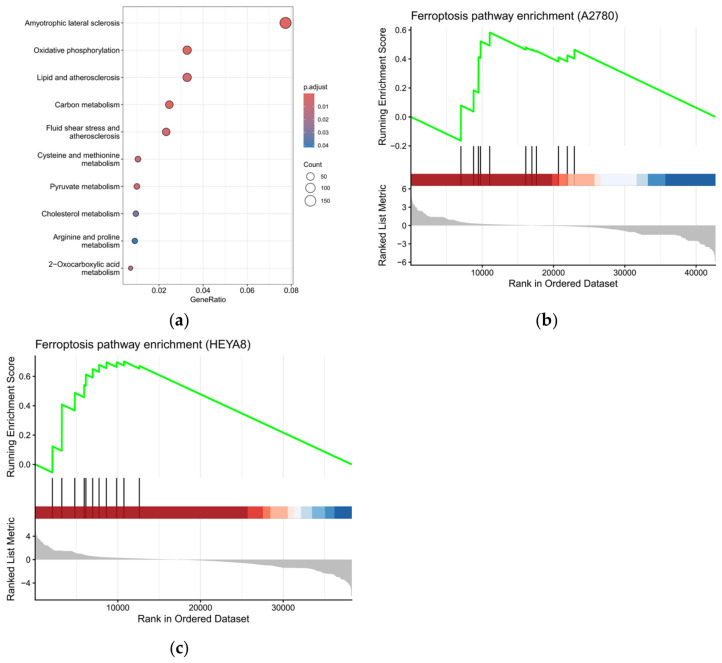
Pathway and gene set enrichment analyses reveal activation of ferroptosis-related programs. (**a**) KEGG pathway enrichment analysis of genes showing transcriptional changes following PunA treatment. Enriched pathways include oxidative phosphorylation, lipid metabolism–related pathways, and cysteine and methionine metabolism. These pathways are associated with mitochondrial function, lipid metabolism, and redox regulation, which are processes commonly linked to ferroptosis. Given the use of technical replicates, pathway enrichment results are interpreted as exploratory and indicative of functional trends rather than definitive statistical conclusions. (**b**,**c**) Gene set enrichment analysis (GSEA) of ferroptosis-related genes in A2780 (**b**) and HEYA8 (**c**) cells. GSEA was performed using ranked gene expression data to evaluate coordinated transcriptional changes associated with ferroptosis. In A2780 cells, a positive enrichment trend of ferroptosis-related genes was observed, indicating a shift toward ferroptosis-associated transcriptional patterns. In HEYA8 cells, a stronger enrichment was detected (NES = 1.80, adjusted *p* = 0.01), suggesting a more pronounced activation of ferroptosis-related gene expression programs. Leading-edge genes include key regulators of ferroptosis, such as *GPX4*, *SLC7A11*, *TFRC*, *NQO1*, and *HMOX1*, reflecting coordinated changes in redox balance, lipid metabolism, and iron homeostasis.

To gain pathway-level insight, KEGG pathway enrichment analysis was performed using differentially expressed genes in cancer cells. Significantly enriched pathways included oxidative phosphorylation, lipid metabolism–related pathways, and cysteine and methionine metabolism (Figure 9a). These pathways reflect alterations in mitochondrial function, lipid peroxidation, and glutathione metabolism, respectively, all of which are closely linked to ferroptosis. In addition, several neurodegenerative disease–related pathways were enriched, which are primarily driven by mitochondrial dysfunction and oxidative stress rather than disease-specific processes.

To assess whether ferroptosis-related pathways are globally activated, gene set enrichment analysis (GSEA) was performed using a curated ferroptosis gene set (Figure 9b,c). In A2780 cells, a positive enrichment trend of ferroptosis-related genes was observed, indicating activation of ferroptosis-associated transcriptional patterns. In HEYA8 cells, enrichment was more pronounced (NES = 1.80, adjusted *p* = 0.01), suggesting a stronger activation of ferroptosis-related gene expression programs. Leading-edge genes included key regulators such as *GPX4*, *SLC7A11*, *TFRC*, *NQO1*, and *HMOX1*, representing coordinated changes in redox balance, lipid metabolism, and iron homeostasis.

Consistent with these findings, global analysis across cancer cells revealed a positive enrichment trend of ferroptosis-associated genes (NES = 1.43, adjusted *p* = 0.096), although statistical significance was modest. Nevertheless, the consistent upregulation of core ferroptosis regulators supports activation of ferroptosis-associated transcriptional programs.

Together, these results demonstrate that PunA likely induces a coordinated ferroptosis-associated transcriptional program that is preferentially activated in cancer cells, with HEYA8 cells exhibiting the strongest response.

## 4. Discussion

Our findings indicate that the omega-5 fatty acid PunA suppresses OC cell growth in vitro and shows preliminary evidence of activity in vivo. Notably, PunA preferentially reduced the viability of OC cells while showing limited effects on normal epithelial cells, suggesting differential sensitivity. Mechanistically, PunA was associated with coordinated changes in lipid metabolism and redox homeostasis, including increased lipid peroxidation, modulation of PPAR-γ, altered FAO, and impaired mitochondrial dysfunction, alongside transcriptional reprogramming associated with oxidative stress and ferroptosis-related pathways. Together, these data support a model in which PunA engages lipid-dependent vulnerabilities in OC cells, potentially converging on ferroptotic cell death While these findings highlight therapeutic potential, further in vivo validation is required.

Building on our prior work in immunodeficient models [13], we evaluated PunA in an immunocompetent C57BL/6 mouse model to better capture tumor-immune interactions. Consistent with earlier observations, aged hosts supported greater tumor growth than younger animals (Figure 1). PunA treatment was associated with reduced tumor burden; however, interpretation is limited by small sample size (Figure 2). In vitro, PunA selectively reduced the viability of OC cells while sparing NFT epithelial cells, whereas its structural isomer α-ESA was broadly cytotoxic (Figure 3). Given that NFT cells are believed to give rise to the majority (>80%) of HGSOC—the most common and most lethal subtype of OC [40]—this preferential cytotoxicity may be highly relevant for therapeutic development, as many current therapies lack tumor specificity and are limited by off-target toxicity [41]. These findings extend prior reports of PunA activity in other cancer types [18,19] and support further investigation of its effects on OC.

The distinct toxicity profiles of PunA and its isomer α-ESA underscore the importance of stereochemistry in dictating biological activity. A plausible explanation for their divergent effects is that the two FFAs are metabolized and incorporated differently into cellular membranes. Compared to NFT cells, OC cells typically express higher levels of acyl-CoA synthetase long-chain family member 4 (ACSL4) [42], an enzyme that esterifies polyunsaturated fatty acids into phospholipids [43]. The 13-cis bond geometry of PunA may make it a preferred substrate for ACSL4, thus making it more likely to be incorporated into the phospholipid bilayer. Once integrated into the bilayer, PunA-containing phospholipids become highly susceptible to lipid peroxidation, a process that can compromise membrane integrity and promote cell death [44]. In contrast, the 13-trans configuration of α-ESA may reduce its suitability as an ACSL4 substrate, limiting its incorporation into membrane phospholipids. Without efficient integration, α-ESA would generate far less oxidative membrane damage and is more likely to initiate a more general, non-selective cell death mechanism, such as apoptosis. This model is consistent with the observed selectivity but remains speculative and requires direct validation.

Furthermore, PunA-induced lipid peroxidation aligns with ferroptosis, an iron-dependent form of cell death that is mechanistically distinct from apoptosis and from the action of platinum-based agents such as cisplatin [45,46]. This distinction prompted us to evaluate whether PunA could enhance cisplatin efficacy (Figure 4). In HEYA8 cells, PunA reduced cell viability and increased cytotoxicity when combined with cisplatin, although the combination did not significantly exceed the effect of PunA alone. HSA analysis was consistent with a cooperative interaction between PunA and cisplatin, as the combined effect exceeded that of the most active single agent [47]. However, this interpretation is model-dependent, and additional analyses using complementary frameworks will be required to more definitively characterize the interaction. Collectively, these findings support the potential of PunA as an adjunct to cisplatin-based therapy in OC.

To assess clinical relevance, we next examined PunA activity in settings of cisplatin resistance and metastatic progression (Figure 4) [48]. In cisplatin-sensitive and -resistant cell lines, PunA reduced viability in both groups, although resistant cells were less sensitive. While this indicates retained activity, the lack of enhanced efficacy in resistant cells suggests that PunA is unlikely be effective as a standalone therapy for cisplatin-resistant OC but may still have value as part of combination strategies in these contexts.

Metastatic spread into the adipose-rich omentum represents a major clinical challenge in OC and is partly driven by interactions between cancer cells and adipocytes [49,50]. To assess whether this lipid-rich environment influences PunA activity, HEYA8 cells were cultured in adipocyte-conditioned medium.

PunA reduced cell viability in a dose-dependent manner under both conditions; however, cells exposed to adipocyte-conditioned medium exhibited reduced sensitivity compared to those cultured in control conditions. These observations suggest that adipocyte-derived factors can attenuate the cytotoxic effects of PunA, highlighting the modulatory role of the lipid-rich microenvironment. The reduced sensitivity to PunA observed under adipocyte-conditioned conditions suggests that the cellular response to treatment is significantly influenced by the metabolic context. This decreased responsiveness may be attributed to metabolic reprogramming induced by adipocyte-derived components, including fatty acids and adipokines. These factors have been reported to support tumor cell metabolism by providing energy substrates and enhancing metabolic flexibility, thereby promoting cellular adaptation and survival under stress conditions [49]. Furthermore, the significant interaction between PunA treatment and conditioned medium indicates that its anti-tumor activity is context dependent. In this setting, the effect of PunA should be interpreted not only based on its intrinsic cytotoxic properties, but also in relation to the surrounding microenvironment shaped by adipocyte-derived signals. Together, these observations highlight the importance of considering metabolic context when evaluating therapeutic responses in OC.

We then investigated direct evidence supporting ferroptosis as the underlying mechanism (Figure 5). Following PunA treatment, expression of ferroptosis-related genes increased; additionally, pharmacological inhibition of ferroptosis in PunA-treated cells restored cell viability, supporting pathway involvement. Importantly, ferroptosis is distinct from apoptosis and other forms of regulated cell death [51]. Consistently, BODIPY 581/591 C11 assays showed a pronounced red-to-green fluorescence shift in HEYA8 cells after PunA treatment, with a 16.1% net increase in Q-positive cells, indicating substantial accumulation of oxidized lipids. In contrast, the P201 cells showed only a 0.2% net increase in Q-positive cells, reflecting minimal levels of oxidized lipids. No comparable oxidative effects were observed in untreated or vehicle (methanol)-treated controls, suggesting that these changes are specific to PunA exposure. These findings support a model in which PunA incorporation into cancer cell membrane phospholipids enhances susceptibility to iron-dependent lipid peroxidation and ferroptosis.

To further explore mechanistic specificity, we examined whether PunA engages key metabolic regulators (Figure 6). PunA upregulated PPAR-γ, a lipid-sensing nuclear receptor implicated in metabolic control and tumor suppression [52], in OC cells but not in normal epithelial cells, suggesting differential activation of lipid-sensing pathways. PPAR-γ is known to regulate lipid metabolism, mitochondrial function, and tumor biology [52,53]. Treatment with omega-3’s has been shown to suppress tumor growth while increasing PPAR-γ expression [54]. These observations raise the possibility that PunA may act, at least in part, through PPAR-γ activation, altering lipid homeostasis in a way that enhances susceptibility to ferroptosis. This is consistent with prior evidence that PPAR-γ regulates genes involved in proliferation and inflammation [55], providing a plausible transcriptional link between PunA treatment and an anticancer phenotype.

However, PPAR-γ’s effects are highly ligand-dependent, and some synthetic ligands suppress OC proliferation through PPAR-γ-independent mechanisms [56]. Thus, PunA’s anticancer activity is unlikely to be explained by PPAR-γ signaling alone. Instead, our collective data suggests that PunA acts through a combination of mechanisms, modulating PPAR-γ while simultaneously promoting the lipid peroxidation that drives ferroptosis. Considering these activities are observed in OC cells but not normal cells, the proposed integrated metabolic and oxidative model may account for PunA’s observed preferential toxicity. Further studies are needed to define how PPAR-γ and PunA can be jointly leveraged in OC treatment.

Building on these observations of differential metabolic signaling, we next examined whether PunA also alters FAO-related signaling. PunA treatment increased HADHA expression in A2780 and HEYA8 cells but not in P201 or E7 cells, indicating enhanced FAO activity in OC cells relative to normal cells. While FAO can support tumor growth and survival [38], increased reliance on this pathway may also elevate oxidative stress, thereby sensitizing cells to ferroptosis. This interpretation aligns with our earlier findings on lipid peroxidation and PPAR-γ modulation.

Because FAO is closely linked to mitochondrial function, we next examined whether PunA also affects broader mitochondrial function (Figure 7). Seahorse XF analysis revealed that PunA significantly impaired mitochondrial respiration in HEYA8 cells by reducing basal and maximal respiration, ATP production, and spare respiratory capacity. This signals that PunA disrupts oxidative phosphorylation in OC cells, limiting their ability to meet energy demands and potentially amplifying oxidative stress that drives ferroptosis. Conversely, P201 cells exhibited minimal changes in mitochondrial function under the same conditions, mirroring their resistance to PunA-induced lipid peroxidation and ferroptosis. This selective preservation of mitochondrial activity suggests that normal cells maintain redox balance and mitochondrial integrity despite PunA exposure. These findings position mitochondria as the potential central mediator of PunA’s preferential toxicity towards cancerous cells. Together with FAO alterations, this supports a model in which metabolic reprogramming contributes to ferroptotic vulnerability.

Finally, we explored the transcriptional basis of PunA’s preferential activity using RNA-seq analysis (Figure 8 and Figure 9). Transcriptomic profiling revealed a robust and cell type–specific transcriptional response, with cancer cells exhibiting substantially greater changes than normal epithelial cells. Among the cancer cell lines, HEYA8 cells displayed the most pronounced transcriptional shifts, consistent with their heightened sensitivity observed in functional assays. Rather than broadly affecting unrelated signaling pathways, PunA induced coordinated transcriptional changes primarily associated with oxidative stress and ferroptosis. Cancer cells, which typically maintain elevated basal levels of reactive oxygen species (ROS) due to metabolic reprogramming and mitochondrial dysfunction, are particularly vulnerable to additional oxidative perturbations [57]. In this context, the consistent upregulation of redox-regulating genes such as *NQO1* and *TXNRD1* likely reflects a compensatory response to PunA-induced oxidative stress. Importantly, these transcriptional changes extended beyond general oxidative stress and involved coordinated activation of ferroptosis-associated pathways. Consistent with the iron-dependent accumulation of lipid peroxides characteristic of ferroptosis [58,59], PunA induced coordinated upregulation of genes involved in redox regulation, lipid metabolism, and iron handling, which are all key functional axes of this pathway.

Pathway-level analysis further supported this model, revealing enrichment of pathways associated with oxidative phosphorylation, lipid metabolism, and cysteine and methionine metabolism. Notably, the latter is closely linked to glutathione (GSH) synthesis, which plays a central role in maintaining redox balance and regulating ferroptosis through GPX4 activity [60]. In addition, enrichment of neurodegeneration-related pathways likely reflects shared underlying mechanisms, particularly mitochondrial dysfunction and oxidative stress, rather than disease-specific processes [61]. Consistent with these findings, gene set enrichment analysis demonstrated a positive enrichment trend of ferroptosis-related genes, with stronger enrichment observed in HEYA8 cells. Although statistical significance was modest in some comparisons, the coordinated regulation of multiple ferroptosis-associated genes suggests activation of a ferroptotic transcriptional program.

In contrast, normal epithelial cells exhibited relatively limited transcriptional changes following PunA treatment, indicating a greater capacity to maintain redox and metabolic homeostasis. This differential transcriptional responsiveness supports a model in which PunA preferentially perturbs cancer cell–specific vulnerabilities, including oxidative stress imbalance and ferroptosis susceptibility.

Taken together, these transcriptomic findings provide mechanistic support for the observed biological effects of PunA, indicating that its anticancer activity is associated with coordinated reprogramming of pathways involved in redox regulation, metabolism, and ferroptosis, while exerting substantially weaker effects in normal cells.

In summary, our findings suggest that PunA may represent a promising anticancer candidate, potentially driven by its ability to induce ferroptosis in OC cells while sparing normal epithelial cells. Across multiple experimental platforms, PunA consistently affected pathways associated with cancer cell vulnerabilities, including lipid peroxidation, upregulation of PPAR-γ, disruption of mitochondrial respiration, enhancement of FAO-dependent oxidative stress, and transcriptional reprogramming of the pathways associated with redox regulation, metabolism, and ferroptosis. Pertinently, these effects appeared less pronounced in normal epithelial cells. However, interpretation of these findings is limited by the use of immortalized normal cell lines, and further in vivo studies are required to validate these proposed mechanisms. Preliminary in vivo data, although limited in sample size, further suggested that PunA reduces tumor burden in immunocompetent mouse models. Collectively, these findings support the therapeutic potential of PunA and its potential use in combination with existing chemotherapeutic agents. Given that cisplatin remains a cornerstone of OC treatment but is limited by dose-limiting toxicities, our results raise the possibility that combining PunA with cisplatin could enable dose-reduction without compromising efficacy, thereby improving tolerability in clinical settings.

The primary limitation of this study is the limited scope of in vivo validation, which will be essential to fully establish the efficacy, safety, and pharmacokinetic profile of PunA. Existing evidence from both animal and human studies suggests that PunA may be incorporated in vivo but is rapidly metabolized to conjugated linoleic acid (CLA), the predominant form detected in plasma and tissues [62,63]. For example, a randomized controlled trial demonstrated that supplementation with *Trichosanthes kirilowii* seed kernels increased levels of both PunA and CLA in plasma and red blood cell membranes, supporting in vivo conversion [63]. However, key pharmacokinetic parameters—including bioavailability, circulating levels of the parent compound, and metabolic stability—remain insufficiently characterized [62,63,64]. Additionally, CLAs have been reported to exhibit relatively low oxidative stability, suggesting that their biological activity and systemic exposure may be influenced by oxidative conditions [65].

The dosing regimen used in this study was guided by prior literature and experimental feasibility; however, the relationship between administered dose and effective systemic exposure remains unclear due to limited pharmacological data. Accordingly, these findings should be interpreted as exploratory, and further studies are needed to define pharmacologically relevant dosing strategies and exposure–response relationships. Nevertheless, as an omega-5 FFA, PunA may possess a favorable safety profile. Our preliminary animal data indicates that IP administration of PunA is feasible and well tolerated, suggesting a viable route for delivery. Future studies incorporating comprehensive in vivo models and eventual clinical evaluation will be critical to determine the therapeutic utility of PunA, both as a monotherapy and as an adjuvant to platinum-based chemotherapy in OC.

## Data Availability

The original RNA-seq data presented in the study will become openly available in the NCBI Gene Expression Omnibus repository at accession number GSE327747 upon publication.

## Supplementary Materials

The following supporting information can be downloaded at: https://www.mdpi.com/article/10.3390/cells15090792/s1, Table S1: Characteristics of the samples; Table S2: Filtered gene expression matrix; Table S3: Differentially expressed genes between cancer and normal samples; Table S4: Ferroptosis-related differentially expressed genes between cancer and normal samples; Table S5: Differentially expressed genes in A2780 cells; Table S6: Differentially expressed genes in HEYA8 cells; Table S7: Expression changes in key oxidative stress-related genes across cell lines. Figure S1: Volcano plot of differential expression in PunA-treated P201 cells; Figure S2: Volcano plot of differential expression in PunA-treated E7 cells; Figure S3: Magnitude of transcriptional response to PunA treatment across cell types; Figure S4: Expression levels of selected ferroptosis-related genes across different cell lines and treatment conditions.

## Abbreviations

The following abbreviations are used in this manuscript:

OC	Ovarian cancer
FFA	Free fatty acid
PunA	Punicic acid
α-ESA	α-Eleostearic acid
TME	Tumor microenvironment
ECM	Extracellular matrix
AT	Adipose tissue
AME	Adipose tumor microenvironment
CAAs	Cancer-associated adipocytes
NFT	Normal fallopian tube
PPAR-γ	Peroxisome proliferator-activated receptor gamma
IP	Intraperitoneal
HGSOC	High grade serous ovarian cancer
DF	Differentiated
UD	Undifferentiated
Fer-1	Ferrostatin-1
FAO	Fatty acid oxidation
HADHA	Hydroxyacyl-CoA dehydrogenase trifunctional multienzyme subunit alpha
MHC	Major histocompatibility complex
ACSL4	Acyl-CoA synthetase long-chain family member 4
EMT	Epithelial–mesenchymal transition
