# Supplementary material for: Punicic Acid in Ovarian Cancer: Anticancer Activity and Mechanistic Insights
Source: Cells. 2026 Apr 27;15(9):792. doi: 10.3390/cells15090792 (PMC13162863; doi:10.3390/cells15090792)
Supplement: Supplementary file 1 [file cells-15-00792-s001.zip › cells-4165294-Supplementary Figures.pdf]

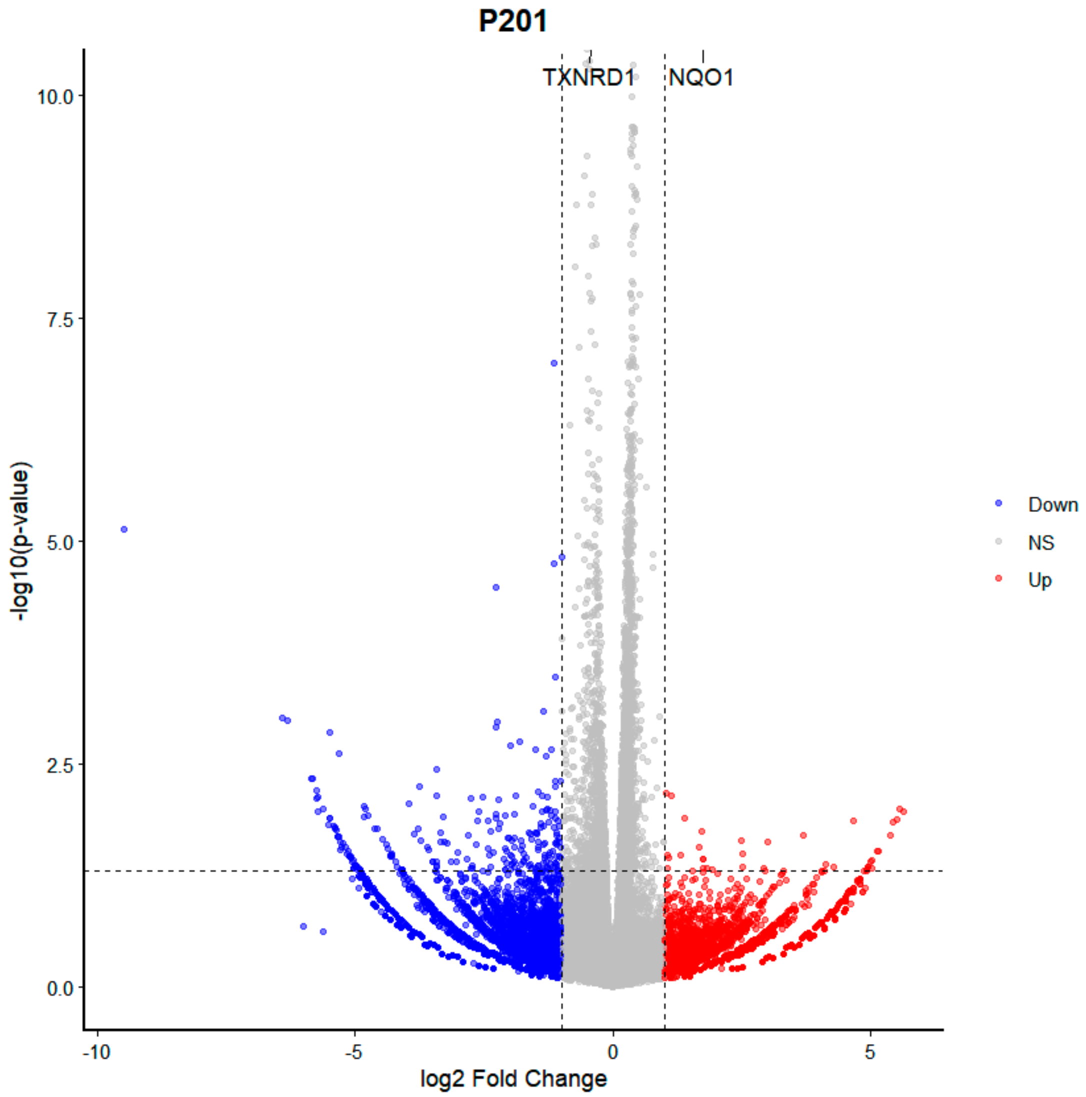

Supplementary Figure S1. Volcano plot of differential expression in PunA-treated P201 cells.

Volcano plot showing gene expression changes in P201 cells following PunA treatment compared with control. The x-axis represents log2 fold change (log2FC), and the y-axis represents −log10 p-value. Genes with fold change greater than 1 (|log2FC| > 0) are highlighted in distinct colors (e.g., red for upregulated and blue for downregulated genes), reflecting differences observed from technical replicates. Other genes are shown in gray.

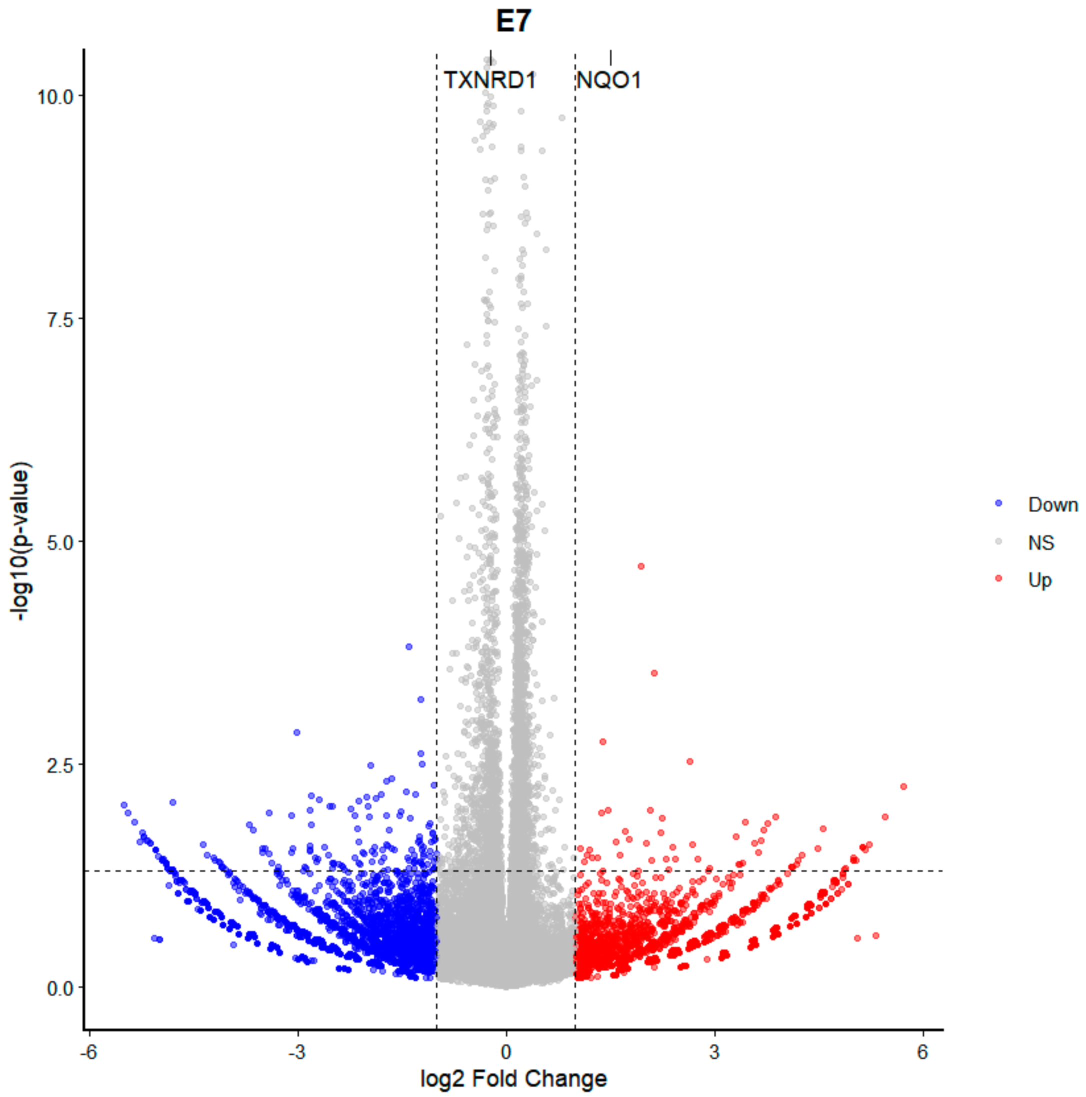

Supplementary Figure S2. Volcano plot of differential expression in PunA-treated E7 cells.

Volcano plot showing gene expression changes in E7 cells following PunA treatment compared with control. The x-axis indicates log2 fold change (log2FC), and the y-axis indicates −log10 p-value. Genes with fold change greater than 1 (|log2FC| > 0) are highlighted in different colors (e.g., red for upregulated and blue for downregulated genes), based on observations from technical replicates, while the remaining genes are displayed in gray.

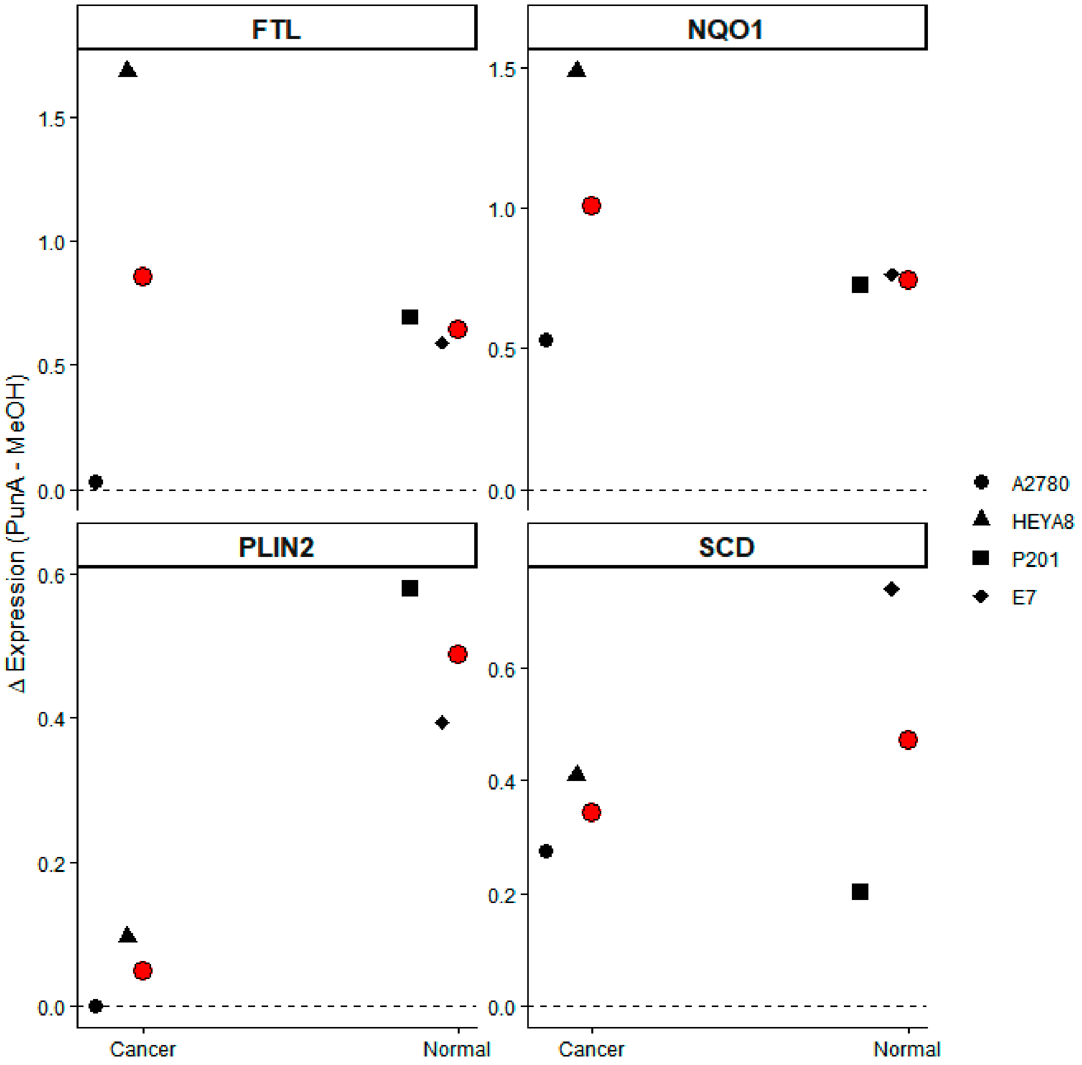

Supplementary Figure S3. Magnitude of transcriptional response to PunA treatment across cell types.

Scatter plots showing the change in gene expression (PunA − MeOH) for selected ferroptosis-related genes in cancer and normal cell lines. Black points represent individual cell lines (A2780, HEYA8, P201, and E7), while red circles indicate the group mean (Cancer vs Normal). Differences in response magnitude between cancer and normal cells are shown to illustrate variability and overall trends in transcriptional responses following PunA treatment.

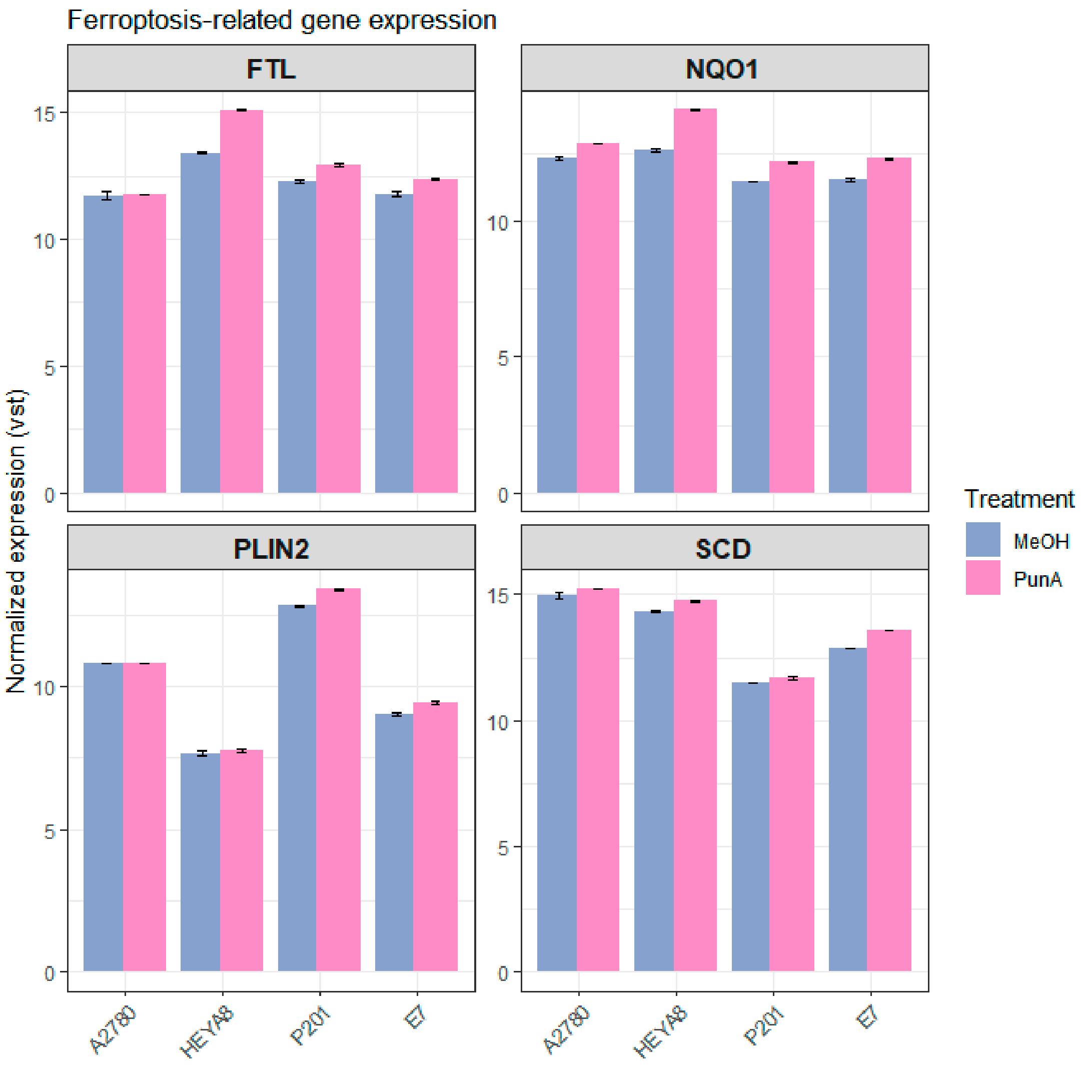

Supplementary Figure S4. Expression levels of selected ferroptosis-related genes across different cell lines and treatment conditions.

Bar plots showing normalized expression levels of representative ferroptosis-related genes across cancer (A2780 and HEYA8) and normal (P201 and E7) cell lines under MeOH control and PunA treatment.

Expression changes are presented to illustrate gene-level transcriptional responses to PunA. Consistent trends are observed for selected genes, supporting the overall transcriptional patterns shown in the main figures.
